# Parietal epithelial cells maintain the epithelial cell continuum forming Bowman's space in focal segmental glomerulosclerosis

**DOI:** 10.1242/dmm.046342

**Published:** 2022-03-14

**Authors:** Laura Miesen, Péter Bándi, Brigith Willemsen, Fieke Mooren, Thiago Strieder, Eva Boldrini, Vedran Drenic, Jennifer Eymael, Roy Wetzels, Johannes Lotz, Nick Weiss, Eric Steenbergen, Toin H. van Kuppevelt, Merijn van Erp, Jeroen van der Laak, Nicole Endlich, Marcus J. Moeller, Jack F. M. Wetzels, Jitske Jansen, Bart Smeets

**Affiliations:** 1 Department of Pathology, Radboud Institute for Molecular Life Sciences, Radboud Institute for Health Sciences, Radboud University Medical Center, 6500HB Nijmegen, The Netherlands; 2 Division of Nephrology and Immunology, University Hospital of the RWTH Aachen, 52074 Aachen, Germany; 3 NIPOKA GmbH, 17489 Greifswald, Germany; 4 Fraunhofer Institute for Digital Medicine MEVIS, 23562 Lübeck, Germany; 5 Department of Biochemistry, Radboud Institute for Molecular Life Sciences, Radboud University Medical Center, 6500HB Nijmegen, the Netherlands; 6 Department of Anatomy and Cell Biology, University Medicine Greifswald, 17489 Greifswald, Germany; 7 Department of Nephrology, Radboud Institute for Health Sciences, Radboud University Medical Center, 6500HB Nijmegen, The Netherlands; 8 Department of Pediatric Nephrology, Radboud Institute for Molecular Life Sciences, Amalia Children's Hospital, 6500HB Nijmegen, The Netherlands

**Keywords:** Parietal epithelial cells, Focal segmental glomerulosclerosis, Epithelial continuum, Bowman's space, Munich Wister Frömter rat

## Abstract

In the glomerulus, Bowman's space is formed by a continuum of glomerular epithelial cells. In focal segmental glomerulosclerosis (FSGS), glomeruli show segmental scarring, a result of activated parietal epithelial cells (PECs) invading the glomerular tuft. The segmental scars interrupt the epithelial continuum. However, non-sclerotic segments seem to be preserved even in glomeruli with advanced lesions. We studied the histology of the segmental pattern in Munich Wistar Frömter rats, a model for secondary FSGS. Our results showed that matrix layers lined with PECs cover the sclerotic lesions. These PECs formed contacts with podocytes of the uninvolved tuft segments, restoring the epithelial continuum. Formed Bowman's spaces were still connected to the tubular system. In biopsies of patients with secondary FSGS, we also detected matrix layers formed by PECs, separating the uninvolved from the sclerotic glomerular segments. PECs have a major role in the formation of glomerulosclerosis; we show here that in FSGS they also restore the glomerular epithelial cell continuum that surrounds Bowman's space. This process may be beneficial and indispensable for glomerular filtration in the uninvolved segments of sclerotic glomeruli.

## Abstract (precis)

**Summary:** Histological analysis of rat and human kidneys reveals a novel role for parietal epithelial cells (PECs) in glomerulosclerosis. PECs seem to restore the glomerular epithelial continuum, which may avert further loss of glomerular function.

## INTRODUCTION

The kidneys are responsible for the removal of waste from our blood, reabsorption of nutrients, and regulation of the body's fluid balance and blood pressure, among other functions. The functional units of the kidneys are the nephrons, consisting of a glomerulus, the filtration unit and a tubular system. In the glomerulus, four main cell types can be identified: endothelial cells, podocytes (visceral epithelial cells), mesangial cells and parietal epithelial cells (PECs). While mesangial cells have a more structural supportive role, endothelial cells and podocytes form together with the glomerular basement membrane the filtration barrier that is responsible for blood filtration and the formation of pro-urine (Arif and Nihalani, 2013). The glomerular capillary tuft is surrounded by Bowman's capsule, creating Bowman's space, into which the filtrate enters after passing through the glomerular filtration barrier. PECs line the Bowman's capsule facing the urinary space. They are connected to the proximal tubular cells at the urinary pole and podocytes at the vascular pole, creating an epithelial cell continuum and ensuring that the pro-urine that is formed is directed to the tubular system.

The glomerulus is vulnerable to injury from inflammatory, metabolic and other disease processes. Injury to the glomerulus can lead to proteinuria (Shankland et al., 2014). Progressive glomerular injury is characterized by the development of focal segmental glomerulosclerosis (FSGS). FSGS may be found without an undefined aetiology; in this case, it is called ‘idiopathic’ or ‘primary’ FSGS. When FSGS develops due to an underlying cause, e.g. hypertension, viral infections, obesity or medications, it is called secondary FSGS (Kuppe et al., 2015; Fogo, 2015). The diagnosis of FSGS indicates that some of the glomeruli appear morphologically normal (focal) and that the affected glomeruli are only partially (segmental) sclerotic (Jennette et al., 2007). The focal and segmental pattern of sclerosis in FSGS distinguishes scarring related to specific diseases from non-specific global sclerosis, affecting the entire tuft, occurring at any age and increasing with ageing (Fogo, 2015). In FSGS, the affected segments of the glomeruli show variable amounts of sclerosis, cellularity, hyaline accumulations and adhesions between the glomerular tuft and the Bowman's capsule.

It is very likely that the functional segments are still perfused and filter, provided that the glomerulus is still connected to a functional proximal tubule. Only the presence of foot-process effacement in the unaffected glomeruli and in the uninvolved (non-sclerotic) segments of sclerotic glomeruli suggests that the structural damage is more widespread than indicated by the histologic lesions (Silva and Pirani, 1988). The segmental development of FSGS depends on local activation of PECs induced by podocyte injury (Smeets et al., 2011; Kriz, 2003; LeHir and Kriz, 2007; Nagata et al., 2017; Nagata and Kriz, 1992). Activated PECs migrate to the capillary tuft and deposit extracellular matrix (ECM), leading to the development of segmental sclerotic lesions (Kuppe et al., 2015; Smeets et al., 2011). It is intriguing that, in kidneys with more advanced disease and older FSGS lesions, the lesions can still appear in a segmental pattern. This suggests that the uninvolved segments of the glomerulus are somehow relatively stable without marked progression of the glomerular lesion.

Glomerular function is tightly regulated and is highly dependent on a correct anatomical configuration, which consists of perfused capillaries accompanied by an intact glomerular filtration barrier that is delimited by Bowman's capsule with a glomerulotubular connection. Also, the ability to develop the necessary pressure equilibrium within the glomerulus is only possible when the glomerular anatomical configuration is intact.

We hypothesize that, in glomeruli with segmental lesions, the anatomical configuration of the uninvolved segments is preserved or restored and functional, which prevents complete loss of the affected glomeruli and explains the segmental appearance of FSGS.

In the present study, we investigated the anatomical configuration of glomeruli with FSGS and question how uninvolved (non-sclerotic) segments are functionally preserved or restored. In particular, we focused on the location and interplay between podocytes and PECs in segmentally sclerotic glomeruli in the Munich Wistar Frömter (MWF) rat model for secondary FSGS, and in human kidney biopsies of patients with secondary FSGS.

## RESULTS

### Matrix layers enclose healthy glomerular segments

**Fig. 1.**
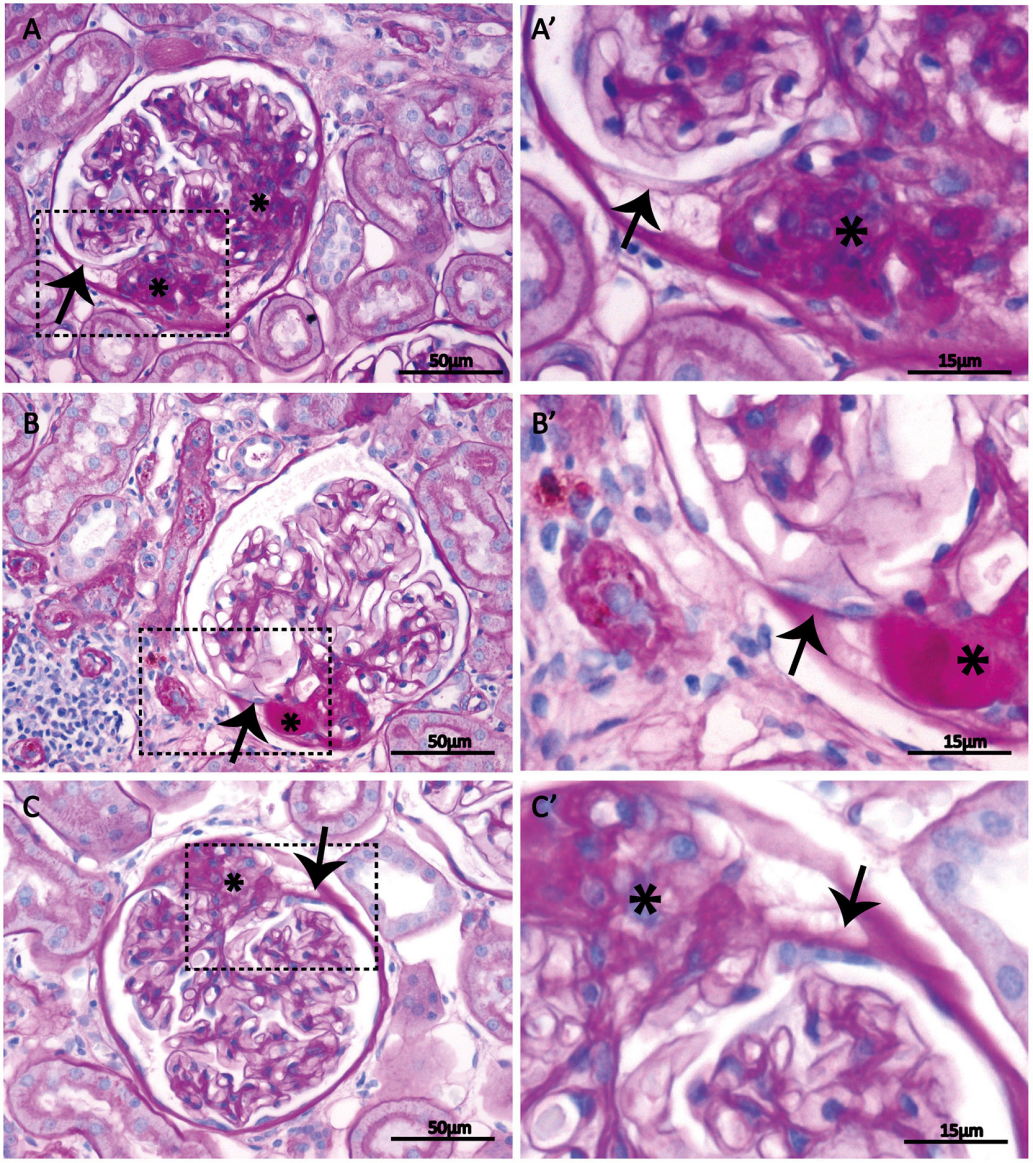
**Matrix layers branching from the original Bowman's capsule line the sclerotic areas of glomeruli.** Kidney tissue slices of MWF rats were periodic acid–Schiff (PAS) stained. (A,B,C) Glomeruli with segmental sclerosis. (A′,B′,C′) Zooms (3×) of the marked areas (rectangles) seen in images A, B and C, receptively. Arrows indicate bifurcations of the original Bowman's capsule. Matrix layers branch off Bowman's capsule and are lined with flat epithelial cells. The matrix layers separate the uninvolved from the sclerotic segments of the glomerular tuft. Asterisks indicate sclerosis.

The histopathology of segmental glomerulosclerotic lesions was studied in kidneys of the MWF rat, a model for secondary FSGS. Most mouse and rat models are very acute, with extensive PEC proliferation in the initial phase and sclerosis development within a few days or weeks, and thus do not mimic the chronic disease development generally seen in many patients. MWF rats have been widely used to study histopathological changes in FSGS because the sclerotic pattern observed in the glomeruli closely resembles the ones seen in human FSGS. We studied the morphology of the sclerotic and the uninvolved glomerular segments, and in particular the boundaries between both segments, in periodic acid–Schiff (PAS)-stained kidney sections from MWF rats with early and advanced FSGS lesions. The FSGS lesions were mostly seen in the perihilar area of the glomerulus. The vast majority of the lesions were segmental, and only in the oldest rats, with the most advanced disease state, were globally sclerotic glomeruli observed. The uninvolved segments of the glomeruli with FSGS showed a normal morphology with open capillaries and some scattered erythrocytes that may imply capillary blood flow at the time of kidney sampling (Fig. S1). These normal-appearing segments seemed to be delimited by Bowman's capsule despite the presence of segmental sclerotic lesions. Closer examination of the Bowman's capsule surrounding the uninvolved segment of the glomerular tufts revealed that the original Bowman's capsule bifurcated into other matrix layers lined by epithelial cells (Fig. 1; Fig. S2). These layers form a connection with the unaffected tuft area, separating the non-sclerotic from the diseased glomerular segments (Fig. 1; Fig. S2).

### PECs form a boundary between sclerotic and uninvolved segments

**Fig. 2.**
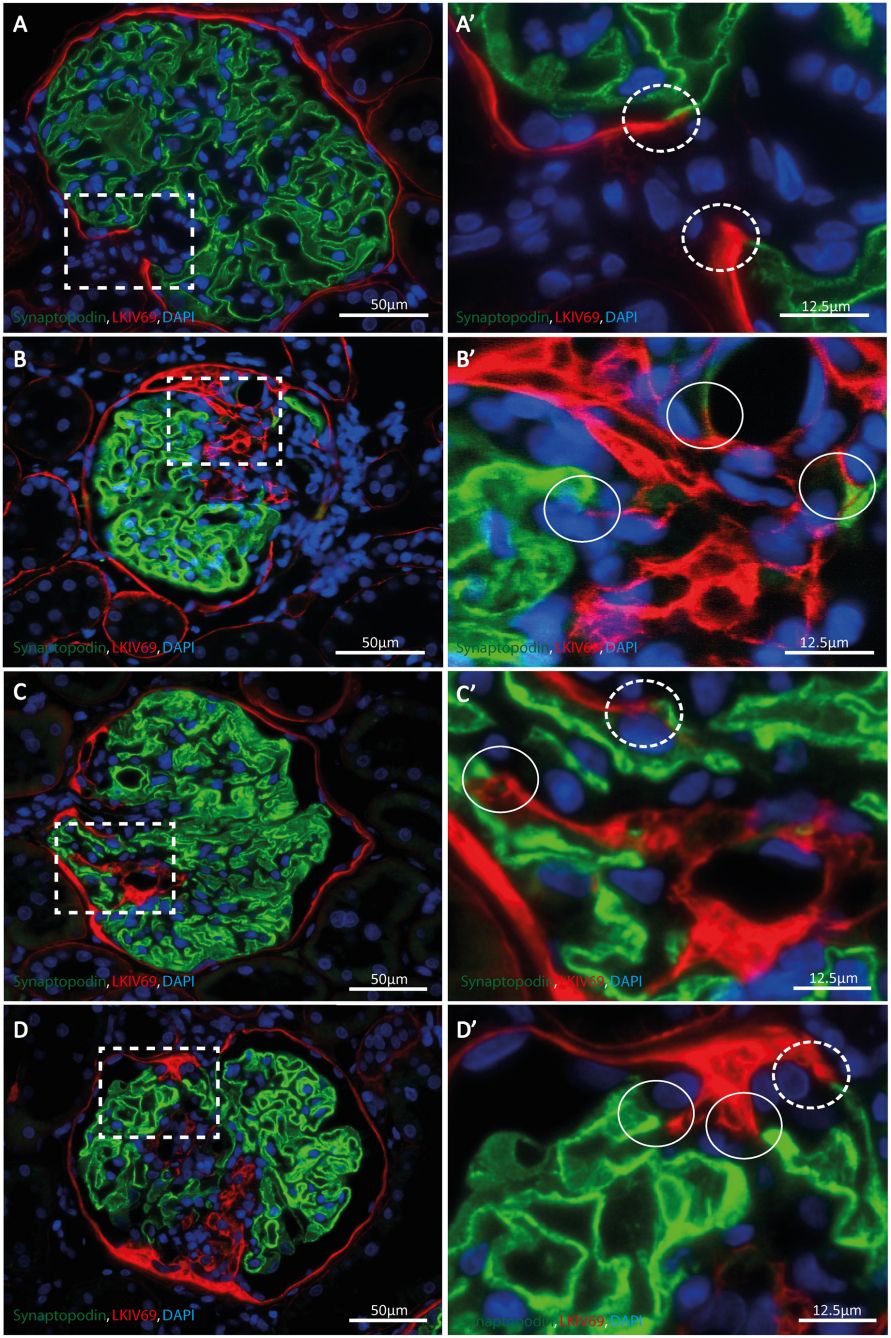
**Connections between parietal epithelial cell (PEC) matrix and the glomerular tuft at sclerotic areas are formed.** Synaptopodin (green), LKIV69 (red) and 4′,6-diamidino-2-phenylindole (DAPI; blue) expression is shown. (A) A non-sclerotic glomerulus of a MWF rat. The glomerular tuft lined with podocytes is connected with the LKIV69-positive Bowman's capsule at the vascular pole, reflecting the connection between PEC matrix and podocytes at this position. (A′) Zoom (4×) of the marked area (rectangle) seen in A. Dashed line circles mark the area of the connection between PEC and podocytes at the vascular pole. (B,C,D) Sclerotic glomeruli of MWF rats containing connections between synaptopodin- and LKIV69-positive matrix at the site of sclerosis. (B′,C′,D′) Zooms (4×) of the marked areas (rectangles) seen in B, C and D, respectively. The circles highlight connections between LKIV69-positive matrix and podocytes at the sclerotic areas. The dashed line circle highlights the connection of the PECs and podocytes at the vascular pole. Single channels are shown in Fig. S4.

**Fig. 3.**
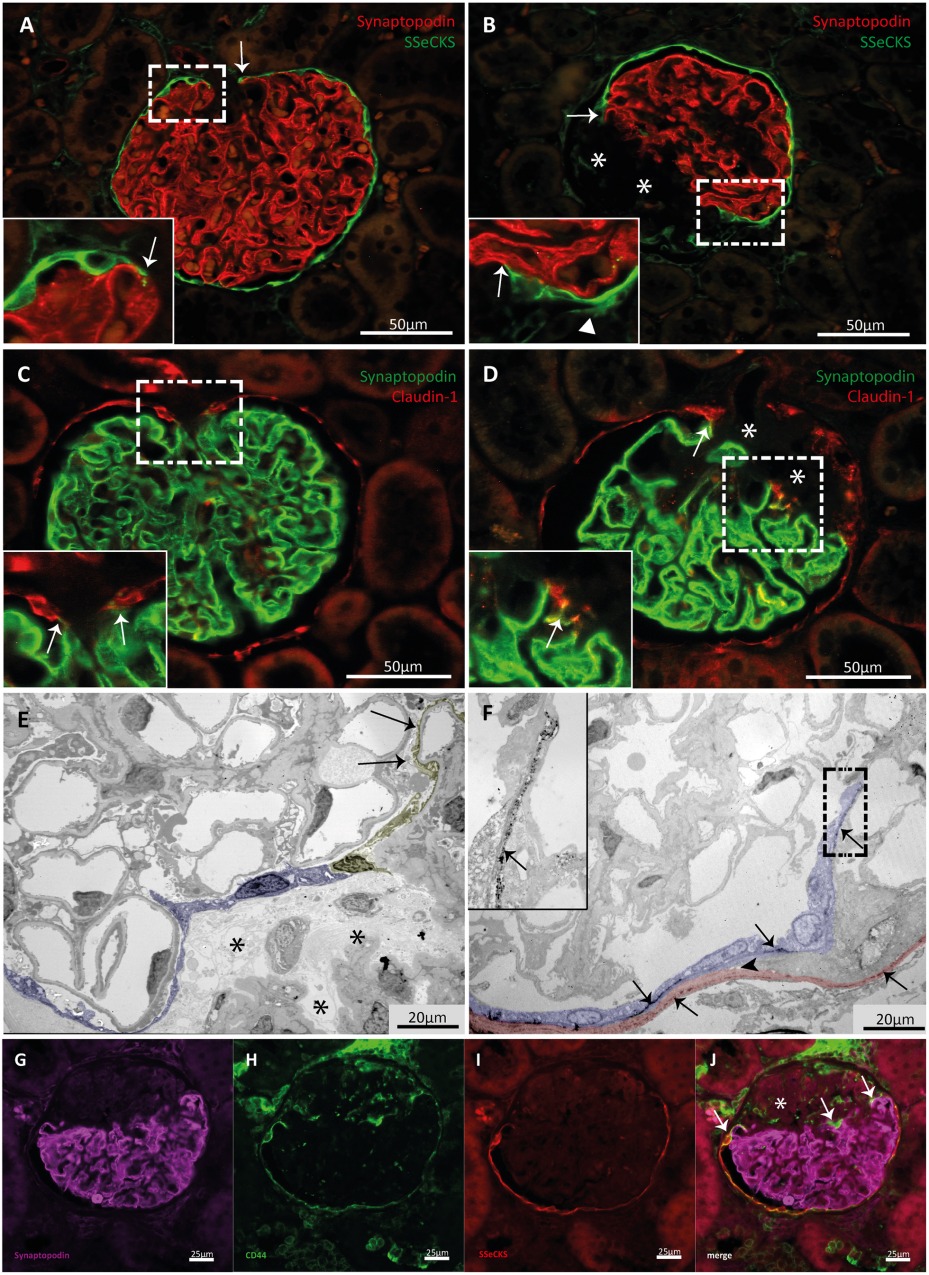
**Connections between CD44-positive PECs and the glomerular tuft at the sclerotic areas are formed.** (A) A non-sclerotic glomerulus of a MWF rat. The glomerular tuft lined with podocytes (red) is connected with the SSeCKS-positive PECs (green) at the vascular pole (arrows), reflecting the connection between PECs and podocytes at this position. (B) A sclerotic glomerulus of a MWF rat is shown, containing connections (arrows) between synaptopodin (red) and SSeCKS (green) expressing cells at the site of sclerosis. The sclerotic area is marked with asterisks. The arrowhead indicates bifurcation of Bowman's capsule lined by PECs. IF images of the single channels are shown in Fig. S5. (C) An image of a healthy glomerulus of a MWF rat is depicted. Claudin-1 signal (red) and synaptopodin signal (green) are in contact at the vascular pole (arrows). (D) A sclerotic glomerulus of a MWF rat is depicted showing contact of claudin-1 (red) and synaptopodin (green) signal at the boundaries of healthy and sclerotic glomerular parts (arrows). Sclerosis is marked with asterisks. Immunofluorescent (IF) images of the single channels are shown in Fig. S6. (E) Electron micrographs, showing a sclerotic (asterisks) and uninvolved segment of a glomerulus. PECs (blue) form a border between the sclerosed capillaries (asterisks) and the healthy glomerular segment. The PECs line a thin matrix layer, which covers loose material within the sclerosed segment. The last PEC is positioned against a podocyte (yellow), identified by the presence of foot processes (arrows). (F) Electron micrograph, showing a sclerotic glomerulus with bifurcation of the Bowman's capsule (arrowhead). LKIV69 immunostaining was examined by indirect immunoelectron microscopy (black granular staining, arrows). Red indicates the original Bowman's capsule. Violet highlights the branching of the capsule and PECs located on LKIV69-positive matrix (inset, zoom). (G-J) PECs that make new connections with podocytes express CD44 (green; H); synaptopodin expression is depicted in violet (G); SSeCKS expression is depicted in red (I); PECs that make new connections with the glomerular tuft are CD44 positive (arrows, J). Sclerosis is marked with an asterisk.

The cells that are present on the matrix layer branching from the original Bowman's capsule have a PEC-like phenotype as they have a flat cell body with cytoplasm that is hardly visible (Fig. 1; Fig. S2). We performed immunofluorescent (IF) staining to investigate the cellular composition at this particular region. Double IF staining for either LKIV69, a marker specific for matrix produced by PECs (Smeets et al., 2014) (Fig. S3), or SSeCKS (also known as AKAP12) or claudin-1, markers for PECs (Burnworth et al., 2012; Colvin and Chang, 2019), and synaptopodin, a marker for podocytes, revealed that the matrix layers covering the sclerotic areas were indeed formed and lined by PECs (Fig. 2, Fig. 3A-D). In addition, we observed that these matrix layers were connected to the glomerular tuft that is lined with synaptopodin-positive podocytes. Notably, these connections resemble the connections between PECs and podocytes at the hilum of the glomerulus (Fig. 2, Fig. 3A-D).

Analysis of the ultrastructure by electron microscopy showed thin epithelial cells layers consisting of PECs extending from Bowman's capsule. These cell layers covered the sclerotic segments of the glomerulus and formed a connection between Bowman's capsule and podocytes of the healthy segments of the glomerular tuft (Fig. 3E). LKIV69 immunostaining was examined by indirect immunoelectron microscopy. Bifurcation of Bowman's capsule could be detected. PECs located on a LKIV69-positive matrix layer, branching from the original capsule, were identified (Fig. 3F).

### PECs that form the connection with the podocytes express CD44

Previous studies by us and others have shown that PECs are involved in the development of FSGS lesions (Dijkman et al., 2005; Kuppe et al., 2015; Smeets et al., 2011; Shankland et al., 2014). These PECs are hypertrophic with enlarged cell bodies and nuclei. In addition, these activated PECs show *de novo* expression of the glycoprotein CD44 (Eymael et al., 2018; Fatima et al., 2012). This phenotype is different from that of the PECs lining the matrix layers covering the sclerotic lesions of affected glomeruli, as these PECs resemble normal quiescent PECs of Bowman's capsule. Nevertheless, IF staining for CD44, SSeCKS and synaptopodin revealed that the flat PECs that form the new matrix layer between the sclerotic and uninvolved glomerular segments are CD44 positive (Fig. 3G-J). This was true for all sclerotic glomeruli we scored in the tissue slices of the rats (*n*=4). In addition, in some glomeruli, flat PECs of the original Bowman's capsule were CD44 positive.

### Bowman's spaces of the uninvolved segments of sclerotic glomeruli are still connected to the proximal tubules

**Fig. 4:**
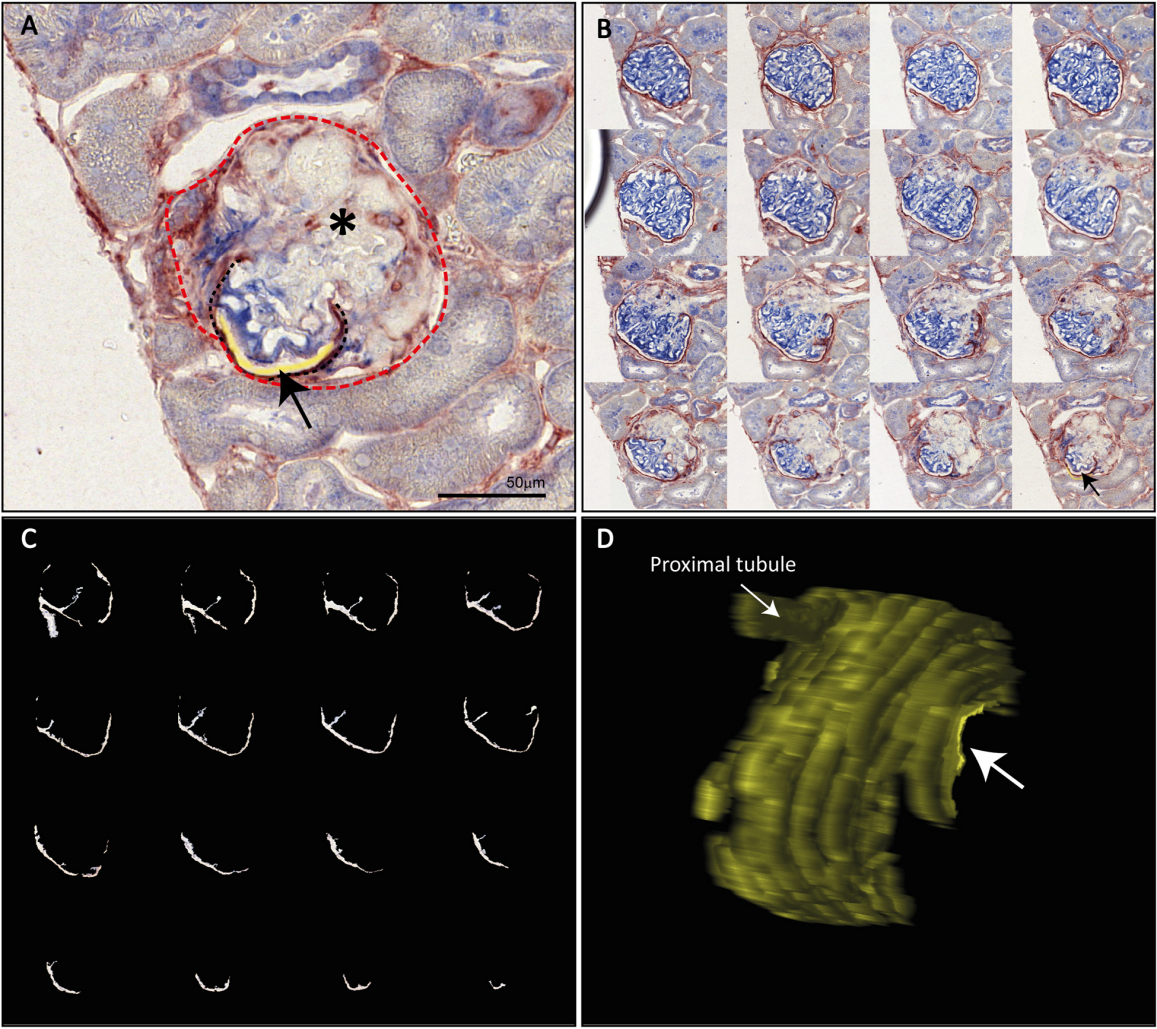
**. Restored Bowman's space is connected to the proximal tubule.** Example of the 3D construction of Bowman's space. (A) Kidney slices are stained for PECs (claudin-1 and SSeCKS, red) and podocytes (nephrin, blue). A sclerotic glomerulus is depicted. The red dashed line indicates the original position of the PECs. Owing to sclerosis (asterisk), the Bowman's space (yellow, arrow) is altered and the PECs surround a non-sclerotic segment of the glomerular tuft (black dashed line). (B) Serial images of the sclerotic glomerulus shown in A. The images show different amounts of sclerosis. The image in the lower right corner is the same as that shown in A. The arrow indicates Bowman's space. (C) Binary masks of the annotated Bowman's spaces shown in the images in B. (D) 3D reconstruction of Bowman's space of the binary masks shown in C, revealing that the altered Bowman's space depicted in the glomerulus in A (arrow) is still connected to the rest of the Bowman's space and to the proximal tubule.

The glomerulus can only filter the blood if it is connected to a tubular system. Although, we observed that Bowman's spaces surrounding the uninvolved segments of sclerotic glomeruli were created by epithelial connections between PECs and podocytes, we also questioned whether these spaces were connected to the proximal tubules. This means that all spaces in a single glomerulus should be connected to each other and to the proximal tubule. To test this hypothesis, serial sections of the rat kidneys were immunohistochemically stained for the PEC markers claudin-1 and SSeCKS and the podocyte marker nephrin (Fig. 4A,B). All serial sections were scanned and registered. Registration of the sections allowed us to stack the sections (Movies 1-4) and to create three-dimensional (3D) reconstructions. To study the Bowman's spaces created by the restored epithelial continuum, we annotated all spaces within the glomerulus that were surrounded by PECs and podocytes. Three-dimensional images of the Bowman's spaces were created using binary masks (Fig. 4C,D). Analysis of the 3D images enabled us to determine whether the spaces in a glomerulus were connected to each other and to the proximal tubule. In three MWF rats, we annotated at least 35 glomeruli per rat. Glomeruli that were about at least 90% visible in the serial sections were scored. In two rats (both 41 weeks old), 82% and 79% of the scored sclerotic glomeruli had restored Bowman's spaces and were still connected to the proximal tubule. In the third rat (54 weeks old), only 58% of the scored sclerotic glomeruli had Bowman's spaces connected to the proximal tubule. The other glomeruli were often atubular. These results indicate that PECs possibly maintain a functional Bowman's space for the uninvolved glomerular segments, which is connected to the proximal tubule.

### Podocytes in uninvolved segments show normal foot-process morphology

**Fig. 5.**
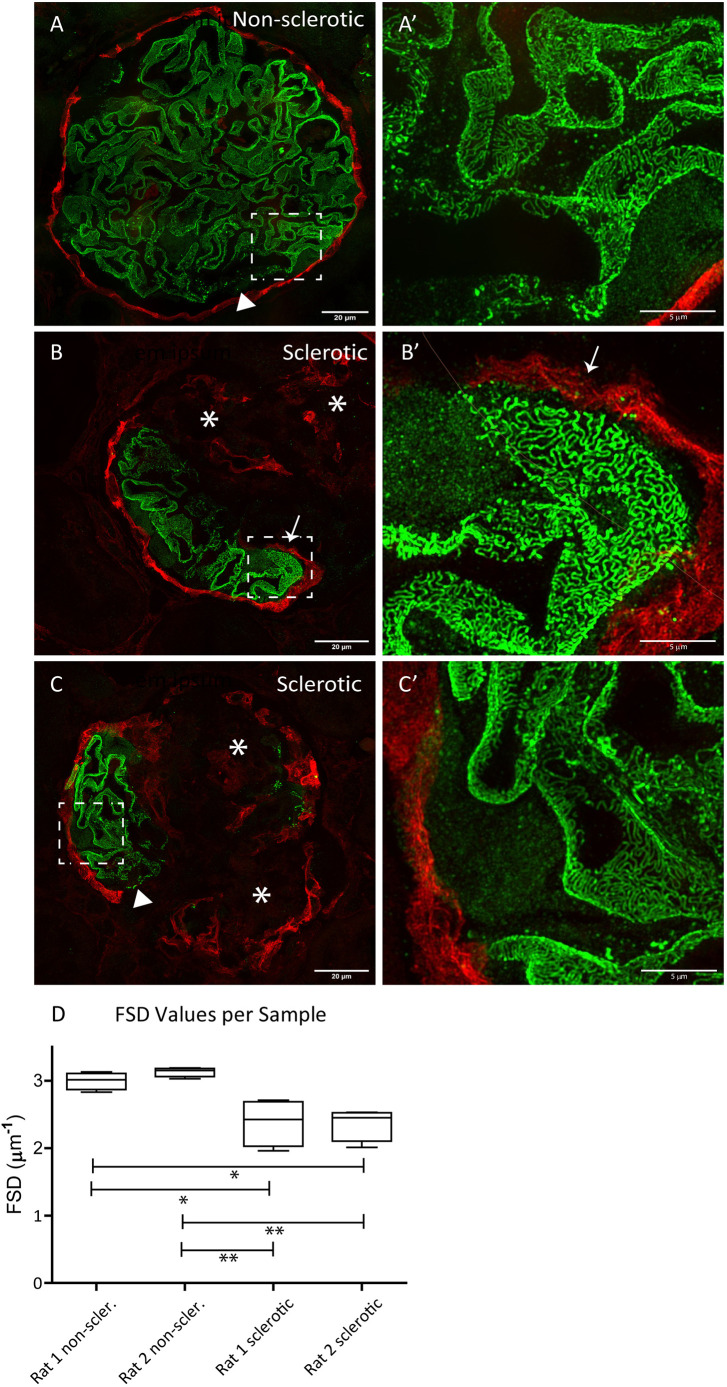
**Uninvolved tuft areas contain podocytes with normal podocyte foot-process morphology.** (A) Super-resolution microscopy image of a non-sclerotic glomerulus in a MWF rat stained for podocin (green) and SSeCKS (red). Podocytes show a dense twisted podocin staining pattern. Some areas showed a disturbed pattern for podocin (arrowhead). (A′) Zoom of the selected area (inset) in A. (B,C) Images of sclerotic glomeruli. The uninvolved (non-sclerotic) segments, surrounded by SSeCKS-positive PECs (B,B′, arrows), show a comparable dense twisted podocin staining pattern as observed in the non-sclerotic glomeruli. Next to areas with normal-appearing foot processes, we also observed some areas with a disturbed pattern (C, arrowhead). (B′,C′) Zooms of the selected areas (insets) in B and C, respectively. (D) Filtration slit density (FSD) measurement by Podocyte Exact Morphology Measurement Procedure (PEMP). The FSD in the uninvolved areas was lower and showed more variation compared to the FSD measured in non-sclerosed glomeruli (*P<0.05, **P<0.01; one-way ANOVA followed by Bonferroni's multiple comparison test).

Next, we studied the morphology of the podocytes at an ultrastructural level. Although we observed normal immunostaining for synaptopodin, we cannot exclude severe foot-process effacement hampering glomerular filtration. To study the foot-process morphology in detail, we used super-resolution microscopy to visualize the foot-process morphology. We focused on podocytes in the uninvolved areas of sclerosed glomeruli that were enclosed by SSeCKS-positive PECs. We compared the podocyte foot-process morphology observed in the uninvolved areas with the morphology observed in non-sclerosed glomeruli of the same kidneys. Large areas in the uninvolved segments showed a normal foot-process morphology (Fig. 5B-C′), which resembled the morphology observed in non-sclerosed glomeruli (Fig. 5A,A′). However, we also observed some areas showing a disturbed staining pattern (Fig. 5C, arrowhead). Such areas were also observed in the non-sclerotic glomeruli (Fig. 5A, arrowhead). Quantitative assessment of the images using Podocyte Exact Morphology Measurement Procedure (PEMP) allowed the quantification of the foot-process morphology by measuring the filtration slit density (FSD). We observed a reduced FSD in the involved segments compared to the non-sclerotic glomeruli, indicating some degree of foot-process effacement compared to the non-sclerotic glomeruli (Fig. 5D).

### PECs form matrix layers between sclerotic and uninvolved segments in human FSGS lesions

**Fig. 6.**
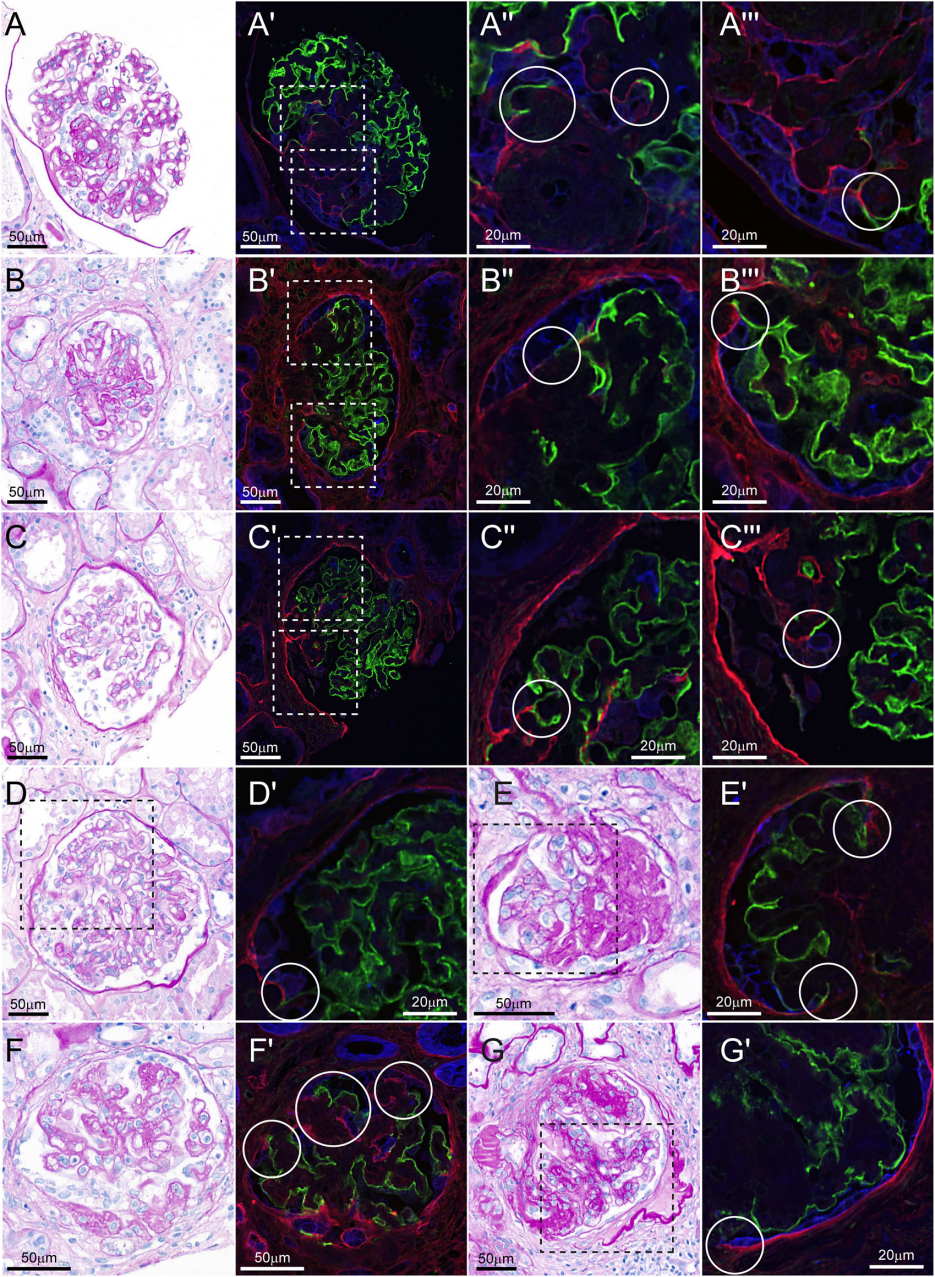
**In humans, PECs and podocytes form connections at the site of sclerosis.** (A-G′) Glomeruli of patients with FSGS lesions are depicted, stained with PAS (A,B,C,D,E,F,G) or for synaptopodin, LKIV69 and ANXA3 (A′-A‴,B′-B‴,C′-C‴,D′,E′,F′,G′). (A-A‴) A glomerulus of a patient suffering from early membranous nephropathy (patient no. 1 of Table 1). (B-B‴) A glomerulus of a patient suffering from chronic thrombotic microangiopathy (patient no. 2 of Table 1). (C-C′″,D-D′) Glomeruli of a patient suffering from monoclonal gammopathy of renal significance (patient no. 3 of Table 1). (E,E′) A glomerulus of a patient suffering from membranous nephropathy (patient no. 4 of Table 1). (F,F′) A glomerulus of a patient suffering from diabetic glomerulosclerosis (patient no. 5 of Table 1). (G,G′) A glomerulus of a patient suffering from diabetic glomerulosclerosis (patient no. 6 of Table 1). Glomeruli shown in PAS staining reflect the same glomeruli shown in IF staining next to the PAS image. Dashed line squares (A′,B′,C′,D,E,G) show areas of zoom. Zoom images are depicted next to the images containing the squares. Circles highlight connections between synaptopodin and LKIV69 and ANXA3 staining signal.

**Fig. 7.**
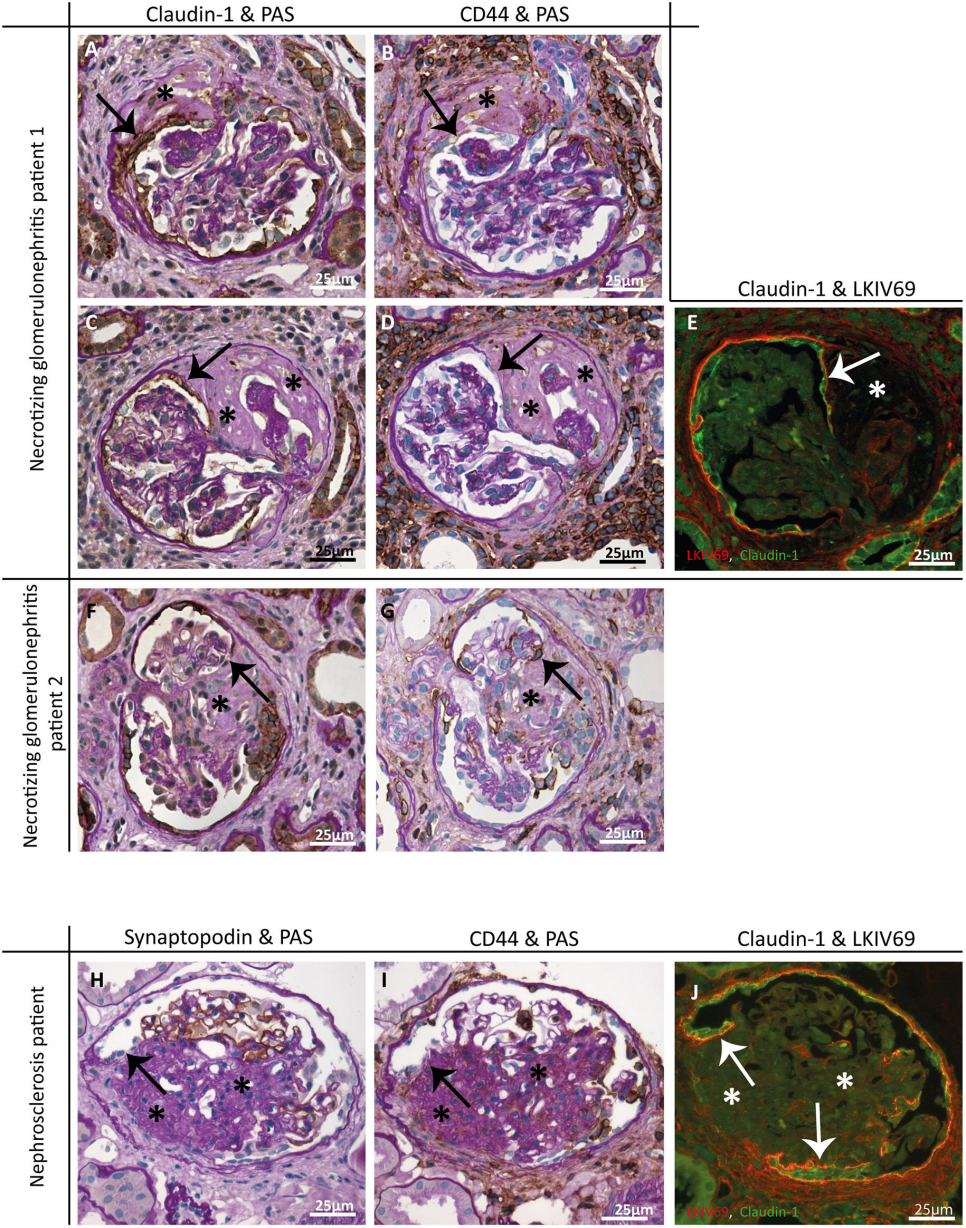
**In humans, CD44-positive PECs form a matrix layer between uninvolved and sclerotic glomerular segments.** (A-G) Glomeruli of two patients suffering from necrotizing glomerulonephritis (patients no. 7 and no. 8 of Table 1). (H-J) A glomerulus of a patient suffering from nephrosclerosis (patient no. 9 of Table 1). (A,C,F) PAS and claudin-1 (brown) staining show that PECs (arrows) separate the sclerotic (asterisks) from the uninvolved segments. (H) PAS and synaptopodin (brown) staining show that small and flat cells line the sclerotic area (arrow) and that these cells are adjacent to functional, synaptopodin-positive podocytes. (E,J) Claudin-1 (green) and LKIV69 (PEC matrix, red) IF staining show that PECs form the matrix layer (arrows), separating the uninvolved and sclerotic (asterisks) glomerular segments. Single channels are presented in Fig. S7. (B,D,G,I) The PECs that form the new matrix layers show CD44 expression (brown, arrows). Asterisks indicate sclerosis.

To translate our findings from the MWF rats to the human situation, we examined kidney biopsies from nine patients with FSGS lesions. In these biopsies, we found glomeruli that resemble the histopathological pattern seen in the MWF rat model (Figs 6 and 7). LKIV69, in combination with ANXA3 and synaptopodin, staining of the biopsies revealed that connections are formed between podocytes and PECs at the site of sclerosis (Fig. 6). PAS, synaptopodin, claudin-1 and LKIV69 staining also showed that newly formed matrix layers had been formed by PECs that lay close to podocytes from the uninvolved segments (Fig. 7A,C,E,F,H,J). Of note, the PECs forming the connection to the podocytes were in most lesions CD44 negative, although in some lesions CD44-positive PECs were observed (Fig. 7B,D,G,I).

**Table 1.**
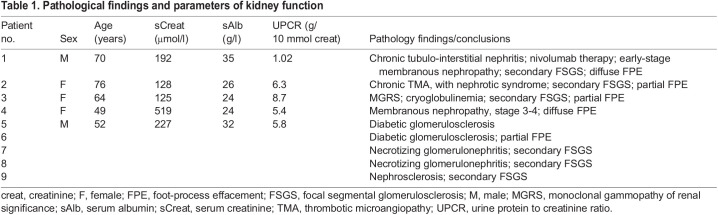
Pathological findings and parameters of kidney function

## DISCUSSION

In this study, we investigated segmental sclerotic lesions in MWF rats, an experimental model for secondary FSGS. We identified that PECs, next to their involvement in the formation of glomerulosclerosis, also participate in the maintenance of the epithelial continuum forming Bowman's space, which is indispensable for glomerular filtration.

**Fig. 8.**
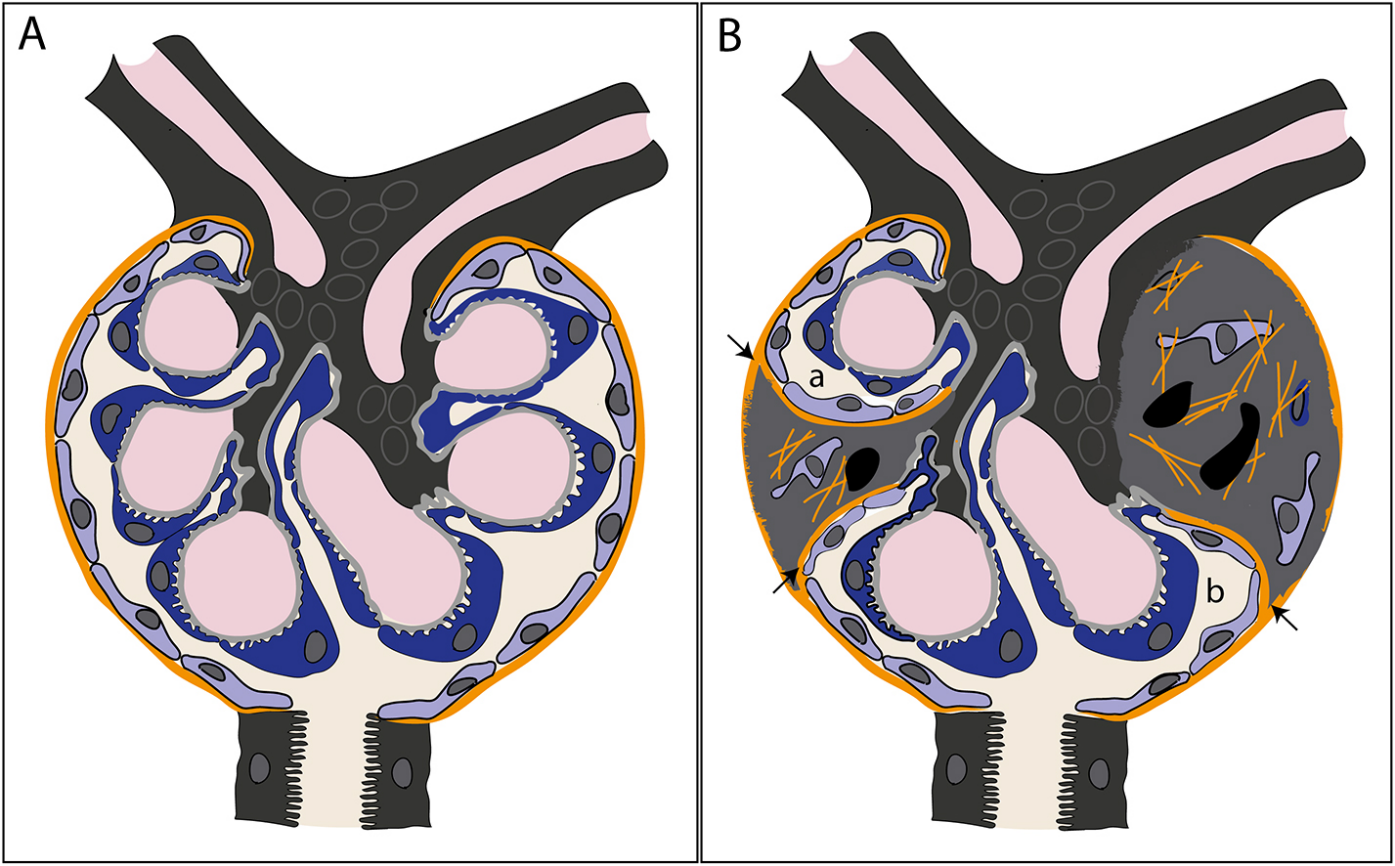
**Schematic illustration of the observations described in this study.** (A) A healthy glomerulus is depicted. PECs line the Bowman's capsule (orange) that surrounds the glomerular tuft and forms the Bowman's space. (B) In segmental sclerotic glomeruli, the uninvolved glomerular segments are still surrounded by a Bowman's capsule (orange) despite the presence of sclerotic lesions (grey). At the borders of the FSGS lesions, the original Bowman's capsule branches off (arrows) and matrix layers, lined by PECs, form a connection between the capsule and the podocytes of the uninvolved tuft segment. Within a single section of a glomerulus, multiple spaces can be formed (a,b), which, in the whole glomerulus (3D), are still connected to each other and to the proximal tubule.

We discovered matrix layers that branch off the original Bowman's capsule, covering sclerotic lesions, and that formed a connection with the remnant glomerular tuft segments. These matrix layers were produced and lined by PECs. The findings are summarized in Fig. 8.

Also, in human biopsies showing secondary FSGS, we detected PECs that form a matrix layer that separates uninvolved from sclerotic glomerular areas and formed connections with the uninvolved tuft segments, indicating that the phenomenon seen in the sclerotic glomeruli of MWF rats occurs in humans as well. In our study, we based our conclusions on findings in secondary FSGS, but whether this process also occurs in primary FSGS has yet to be elucidated.

The role of PECs in FSGS lesions or crescents in rapidly progressive glomerulonephritis has been reported in previous studies (Kuppe et al., 2015; Smeets et al., 2014; Kuppe et al., 2019; Dijkman et al., 2005; Smeets et al., 2011; Gaffney, 1982; Smeets et al., 2009). In these studies, activated PECs are described to be responsible for the formation and progression of sclerotic lesions. Activated PECs proliferate and migrate to the glomerular tuft, while excreting extracellular matrix, which leads to the formation of sclerotic lesions. In the current study, we confirmed the involvement of CD44-positive, activated PECs in sclerosis formation in secondary FSGS. However, we also observed that a subpopulation of PECs is present on matrix layers that branched off the original Bowman's capsule, separating the sclerotic from the uninvolved glomerular segments. An interesting finding was that the morphology of these PECs was similar to that of normal PECs of Bowman's capsule (flat squamous epithelial cells). This phenotype is different from that of activated PECs that have been described to cause glomerulosclerosis, as activated PECs show marked hypertrophy, with enlarged cuboidal cell bodies and enlarged nuclei (Gaffney, 1982). Although PECs that were located on the matrix layers that surround the uninvolved segments of sclerotic glomeruli resemble quiescent PECs, they did express CD44. In a previous study, we showed that CD44 is involved in the proliferation and migration of PECs (Eymael et al., 2018), processes that may also be important for the PECs that cover the matrix layers lining the sclerotic lesions observed in this study.

The PECs described in experiments with the MWF rats appeared not to be a direct part of the glomerular scar but lined the sclerotic lesions and formed connections between the original Bowman's capsule and the uninvolved segments of the glomerular tuft. Similar observations have been described by Kriz et al. (1998). In their study, the sequence of histopathologic events leading from an initial glomerular injury to segmental sclerosis was studied in Fawn-hooded hypertensive (FHH) rats. Similar to the MWF rats used in our study, the lesions were consistently associated with the glomerular vascular pole. In the FHH rats, this was attributed to expansion of primary branches of the afferent arteriole and subsequent podocyte injury and detachment. At the sites of podocyte detachment, PECs form tuft adhesions to Bowman's capsule. Also, in these rats, it was described that PECs demarcated the boundaries between the affected and still-intact tuft remnants (Kriz et al., 1998). However, in the current study, we also focused on the connection between the PECs and the podocytes on the uninvolved tuft segments. The transition between PECs to podocytes at these connections seemed to be similar to the normal transition from PECs to podocytes at the hilum of the glomerulus. Consequently, an epithelial cell continuum and thus an enclosed Bowman's space was created. Restoration of Bowman's space is important for glomerular function because an enclosed Bowman's space is essential for the formation of normal capsular hydrostatic pressures and thus for glomerular filtration. In fact, we observed that the connection between the newly formed matrix layer and the glomerular tuft showed a similar curvature as the original Bowman's capsule, suggesting the presence of capsular hydrostatic pressure. We suppose that, due to the restoration of the Bowman's space, the non-scarred segments can function even when a large part of the glomerulus is sclerotic. We also observed erythrocytes in capillaries in the remaining uninvolved tuft segments, indicating that these segments could still be perfused.

Observing the described phenomenon, the question remains which signalling pathways and cell–cell interactions drive migration of PECs towards the remaining podocytes on the non-sclerotic tuft remnants. In general, local podocyte loss seems to be the basis of segmental sclerosis formation. A local response is initiated to cover the naked glomerular basement membrane (GBM). Podocyte hypertrophy and PECs covering the GBM are possible mechanisms to prevent protein leakage, functioning as wound-healing process (Nagata et al., 2017).

Although the molecular pathways resulting in PEC activation are largely unknown, several signalling routes have been identified to have a possible role in PEC activation and migration of PECs onto the glomerular tuft (e.g. CD44, pERK1/2, mTOR, Wnt-β-catenin, angiotensin II, MIF, CD9 and CXCL12/CXCR4 signalling) (Ito et al., 2020; Lazareth et al., 2019; Miesen et al., 2017; Nagata et al., 2017; Romoli et al., 2018). At this moment, one can only speculate on the sequence of events resulting in the restoration of the epithelial continuum. It is likely that PECs become activated and migrate to the tuft due to chemotactic signals, as described by Ito et al. (2020). Their study showed that injured podocytes increase migration inhibitory factor (MIF) and stromal cell-derived factor 1 (SDF-1, CXCL2) expression that stimulates CD44 expression and CD44-mediated migration in PECs (Ito et al., 2020). PECs migrate along the sclerosed denuded GBM. Next, contact inhibition at the site of podocyte–PEC contacts may result in a stable epithelial cell continuum formed by the PECs and podocytes.

In general, the described process may be important to keep the glomeruli functional, and one may speculate that, in case of remission or successful treatment, the glomeruli remain (partly) functional, without further progression of FSGS. A recent case study by Hamroun et al. (2021) may support this theory. In this particular study, a kidney with recurrence of primary FSGS was retransplanted 8 months after transplantation and reimplanted in another patient. At time of reimplantation, the kidney showed marked FSGS and diffuse podocyte foot-process effacement. In the 4- and 12-month biopsies after reimplantation, the kidney still showed FSGS lesions in over two-thirds of the glomeruli. However, foot-process effacement was reduced and kidney function was restored, indicating that the lesions were inactive and did not affect kidney function (Hamroun et al., 2021).

In conclusion, this study shows that PECs are not only involved in (secondary) FSGS formation but also in the maintenance of the glomerular epithelial cell continuum so that the function of Bowman's space is preserved and the pro-urine created by the uninvolved segments can still be directed to the proximal tubule. To gain more insight into the filtration capacity of the uninvolved segments, perfusion studies using fluorescently labelled tracers should be performed, which would allow the integrity of the restored Bowman's space to be investigated. Another question that remains is whether the PECs that form the epithelial continuum, as described in the current paper, are differentially regulated compared to PECs actively involved in FSGS lesions. This is of importance with respect to the idea to target the processes driving PEC activation and to prevent or attenuate sclerosis. If PECs also fulfil a beneficial role in sclerotic glomeruli, inhibition of, for instance, PEC migration may have adverse effects.

## MATERIALS AND METHODS

### Animal experiments

In the present study, we used MWF rats as a model for secondary FSGS. Initiated by an autosomal gene modification on chromosome 6, MWF rats show a reduced number of nephrons. Spontaneous development of hypertension contributes to injury to intermediate/small vessels and podocytes, which results in progressive glomerular scarring with age and finally renal dysfunction (Hackbarth et al., 1991). After a time period of 13-14 weeks, MWF rats develop proteinuria; FSGS development starts at about 20 weeks of age. For our experiments, only male MWF rats (*n*=8) with an age of 41-54 weeks were biopsied. The rat tissue was intravenously perfused using 3% (w/v) paraformaldehyde, before the kidneys were sampled. The isolated renal tissue was fixed in 4% (w/v) formalin and embedded in paraffin. The rats were obtained from Professor Kreutz, Department of Clinical Pharmacology and Toxicology, Charité Centrum für Therapieforschung, Charité – Universitätsmedizin Berlin, Berlin, Germany (Schulte et al., 2012). All animals were housed under standard conditions. Animal procedures were approved by German government officials (LANUV NRW 50.203.2 – AC 10/06) and performed in accordance with the European Communities Council Directive (86/609/EEC).

### Patient biopsies

Archived kidney biopsies were selected with consent from the local ethics board of the Radboud University Medical Center (file number 2018-4563). Available patient data are provided in Table 1. Patients 6 to 9 were analysed for renal pathology consultation, but were not treated in the Radboud University Medical Center. Clinical data on these four patients are not available.

### Staining methods

Staining was performed on formalin-fixed and paraffin-embedded 4 µm tissue sections. The histopathology was analysed in PAS-stained kidney sections of the MWF rats. PAS staining was performed automatically with a Tissue-Tek Prima™ (Sakura, version 20) histochemical staining machine. In short, slices were deparaffinized, washed with demi water and treated with periodic acid for 10 min. Next, slices were again washed in demi water, incubated for 30 min in Schiff's reagent and rinsed with lukewarm water. After washing in streaming water, slices were incubated with haematin for 10 min and finally washed with demi water.

For IF staining procedures, all antibodies were diluted in PBS-bovine serum albumin (BSA) 1% (v/v). Primary antibodies were incubated either for 1 h at room temperature or overnight at 4°C. Secondary antibodies were incubated for 1 h at room temperature and diluted 1:200. Primary antibodies used are listed in Table S1. The following secondary antibodies were used: Alexa Fluor 488 donkey anti-goat IgG (H+L) (A11055, ThermoFisher Scientific), Alexa Fluor 568 donkey anti-goat IgG (H+L) (A11057, ThermoFisher Scientific), Alexa Fluor 647 donkey anti-mouse IgG (H+L) (ab150107, Abcam), Alexa Fluor 647 donkey anti-rabbit IgG (H+L) (A31573, ThermoFisher Scientific), Alexa Fluor 568 donkey anti-rabbit IgG (H+L) (A10042, ThermoFisher Scientific).

For the detection of the PEC matrix, LKIV69 (1:50; kindly provided by Dr T. van Kuppevelt, Radboud University Medical Center, Nijmegen, The Netherlands) and anti-VSV Glycoprotein−Cy3 (1:400; monoclonal mouse antibody, clone P5D4, C7706, Merck) were used. IF-stained slices were mounted with DAPI Fluoromount-G^®^ (0100-20, SouthernBiotech). IF images were captured using the automated high-content microscope (DMI6000B, Leica Microsystems) or Keyence BZ-9000 Microscope (Keyence Deutschland, Neu-Isenburg, Germany).

Double immunohistochemical (IHC) staining was performed to detect podocytes and PECs on 4 µm consecutive sections of the embedded rat kidney tissue (three series of three different rats). In the double IHC staining, horseradish peroxidase (HRP) and alkaline phosphatase (AP) detection were combined. To avoid non-specific binding of the secondary antibody, slices were blocked with 20% (v/v) normal horse serum. Endogenous biotin, biotin receptors and avidin were blocked with an Avidin/Biotin Blocking Kit (SP-2001, Vector Laboratories). To detect podocytes, slices were incubated with an anti-nephrin antibody (Table S1). As secondary antibody, a horse anti-goat biotinylated antibody was used (1:200; BA-9500, Vector Laboratories). Endogenous peroxidases were blocked with 0.3% (v/v) hydrogen peroxide in PBS. A VECTASTAIN^®^ABC-AP Staining Kit (PK-5000, Vector Laboratories) was used, followed by incubation with the StayBlue/AP solution (ab178453, Abcam), resulting in blue nephrin staining in the podocytes. For the follow-up HRP reaction, slices were blocked with 20% (v/v) goat serum. To ensure optimal staining of PECs, we used two different antibodies directed against the cytoplasmic protein SSeCKS and against the membrane PEC marker protein claudin-1. To detect both PEC makers, we used a Brightvision poly HRP anti-rabbit antibody (VWRKDPVR110HRP, Immunologic). PECs were stained red after incubation with the AEC substrate system (ab64252, Abcam). IHC-stained slices were covered with Fluoromount-G^®^ (0100-01, SouthernBiotech).

Human biopsies were stained with 3,3′-diaminobenzidine (DAB) followed by PAS. In short, endogenous peroxidases were blocked with 3% (v/v) hydrogen peroxide in PBS. Endogenous biotin, biotin receptors and avidin were blocked with the Avidin/Biotin Blocking Kit. Tissue slices were blocked with 20% (v/v) serum. Primary antibodies were used as listed in Table S1. HRP-labelled secondary antibodies against rabbit, mouse or goat were used (Vector Laboratories). Slices were incubated with VECTASTAIN^®^ ABC-HRP Kit, Peroxidase (Standard) (PK-6100, Vector Laboratories) followed by DAB. After dehydration, a PAS stain was applied and slices were mounted.

### Slide registration and 3D reconstruction of Bowman's space

To establish the architecture of the Bowman's capsule and consequently Bowman's space in affected glomeruli, 3D images of the sclerotic glomeruli were made. Using a Pannoramic P250 Flash II tissue scanner (3DHistech, CIS VCC-FC60FR19CL camera, 0.24 µm pixel^−1^) and corresponding computer software (Pannoramic Scanner, version 1.22.0.67865), light microscopic images of the histological slides were digitalized. Each stack of IHC slides was reconstructed by a pairwise registration of neighbouring slides. A stack consisted of a series of images made of the consecutive cuts in every 4 µm from the same paraffin-embedded tissue block. The slide image in the middle of each stack was selected as a starting point and the 3D reconstruction was obtained by concatenating all pairwise results. The pairwise registration is a three-step registration pipeline consisting of a robust pre-alignment, a parametric registration computed on coarse resolution images and a high-resolution nonlinear registration (Lotz et al., 2019 preprint). In all three steps, the normalized gradient fields distance that measures the alignment of image gradients is minimized (Haber and Modersitzki, 2007). In the nonlinear registration, curvature regularization is added to the distance term to favour smooth deformations without foldings (Fischer and Modersitzki, 2003). After registration, glomeruli of interest were selected using the Automated Slide Analysis Platform (ASAP), and serial images of these selected glomeruli were created. Using ImageJ, Bowman's spaces of the single images of one glomerulus were annotated, and binary masks were created to create 3D reconstructions with the ImageJ 3D viewer plugin.

### Super-resolution imaging and filtration slit-density measurements

Sample processing and subsequent imaging was performed as described before (Artelt et al., 2018). In short, 4 µm paraffin sections were directly mounted on coverslips (VWR). To correct for paraformaldehyde-induced autofluorescence, samples were incubated with 100 mM glycine in PBS for 10 min. Samples were blocked with 1% (v/v) fetal bovine serum, 1% (v/v) goat serum, 1% (v/v) bovine albumin and 0.1% (v/v) cold fish gelatin in PBS at room temperature for 1 h. Primary antibodies against nephrin (guinea pig, Progen GmbH, 1:100) and SSeCKS (Table S1; 1:200) were diluted in blocking solution and detected by a secondary anti-guinea pig antibody (1:800) and anti Cy3-labeled polyclonal goat anti-rabbit IgG (H+L) (1:600) (both from Jackson ImmunoResearch, Hamburg, Germany) diluted in blocking solution. Three-dimensional structured illumination microscopy (SIM) images were acquired using a Zeiss Elyra PS.1 system. Using Zeiss ZEN black software, 3D SIM images were reconstructed. Podocyte PEMP was performed using FIJI and a custom-build macro (Siegerist et al., 2017). Analysis was performed in two different MWF rat glomeruli. FSD was measured in eight (four sclerotic and four non-sclerotic) glomeruli.

### Immunoelectron microscopy

LKIV69 immunostaining was examined by indirect immunoelectron microscopy (IEM), using immunoperoxidase labelling on 20 µm frozen sections. One-millimetre-thick kidney slices were immersion fixed in a mixture of 10 mm periodate, 75 mm lysine and 2% (w/v) paraformaldehyde, pH 6.2 (PLP), for 3 h. The slices were washed in PBS for 30 min and cryoprotected by immersion in 2.3 m sucrose solution for 1 h. Finally, tissues were snap-frozen in liquid nitrogen. Cryosections (20 µm) were rinsed in PBS for 1 h and then incubated with the LKIV69 antibody diluted 1:100 in PBS-BSA 1% (v/v) for 18 h at 4°C, followed, after three washes with PBS, by incubation with a secondary antibody anti-VSV diluted in PBS-BSA 1% (v/v). After three washes in PBS, the sections were incubated with a tertiary peroxidase-labelled rabbit anti-mouse antibody diluted in PBS containing 1% BSA. After three washes in PBS, the sections were incubated in PBS, pH 7.4, containing DAB medium for 10 min, followed by DAB with the addition of 0.003% (v/v) hydrogen peroxide for 7 min. The sections were washed in distilled water, post-fixed in Palade buffer containing 1% (w/v) for 30 min at 4°C, dehydrated and embedded in Epon812 (Merck). Ultrathin sections were examined in a JEOL 1200 EX2 electron microscope.

## Supplementary Material

Supplementary information
