# Supplementary material for: Parietal epithelial cells maintain the epithelial cell continuum forming Bowman's space in focal segmental glomerulosclerosis
Source: Dis Model Mech. 2022 Mar 14;15(3):dmm046342. doi: 10.1242/dmm.046342 (PMC8938403; doi:10.1242/dmm.046342)
Supplement: Supplementary information [file dmm-15-046342-s1.pdf]

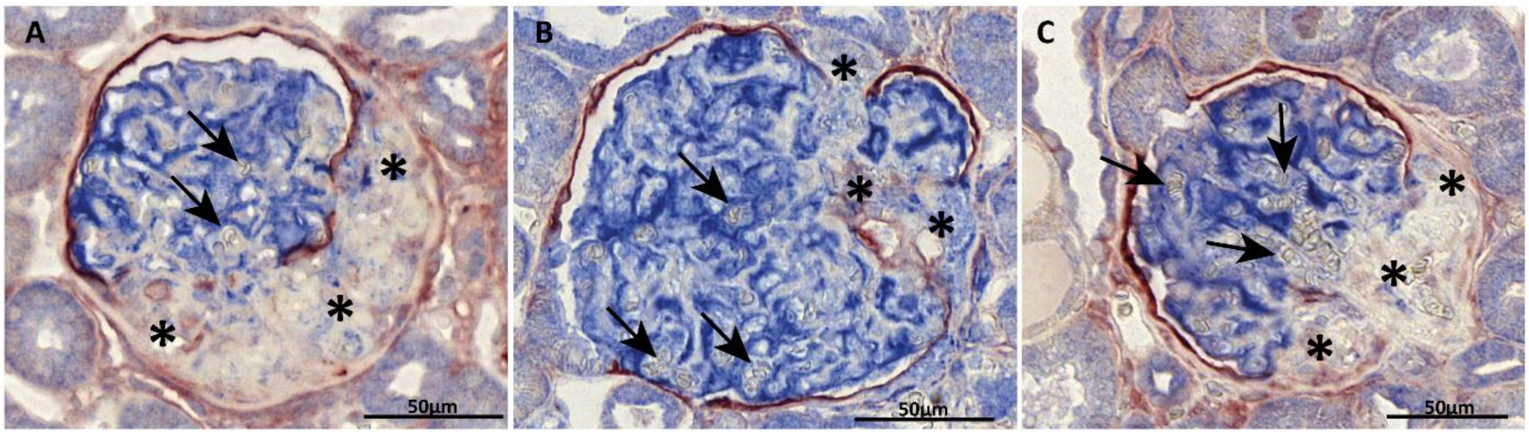

Fig. S1. Erythrocytes are present in non-sclerotic glomerular segments. (**A-C**) Sclerotic glomeruli of MWF rats. Synaptopodin (blue) and SSeCKS and claudin-1 (red) expression is seen. In the non-sclerotic segments synaptopodin expression is preserved. Erythrocytes (arrows) are found in between the podocyte signal, reflecting the capillary lumen. Sclerosis is marked with asterisks.

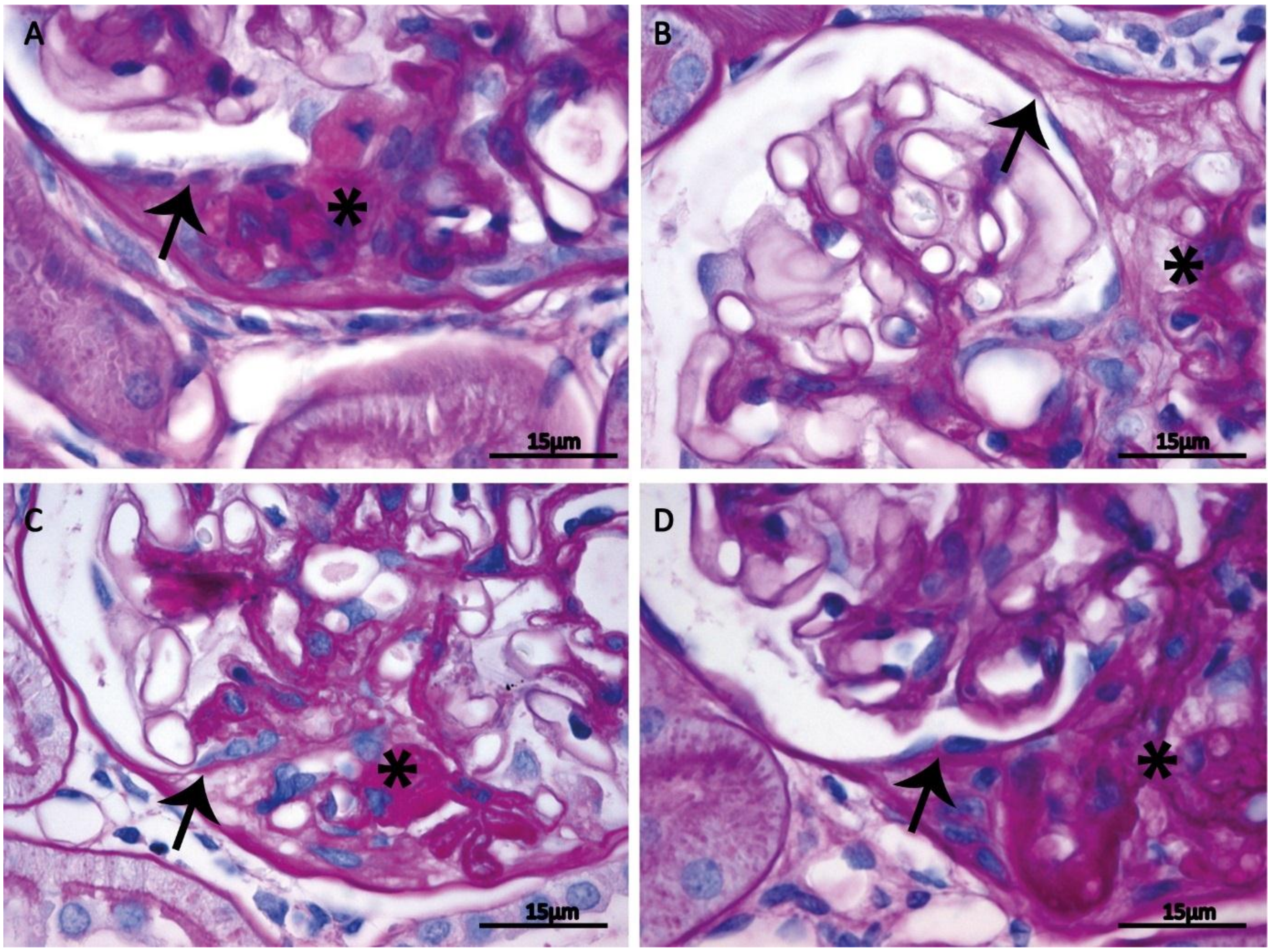

Fig. S2. Matrix layers of the original Bowman's capsule line the sclerotic areas of glomeruli. Kidney tissue slices of MWF rats were PAS stained. (**A**, **B**, **C**, **D**) 4 different glomeruli with segmental sclerosis are depicted. Arrows indicate the newly formed matrix layers lined with cells. Asterisks indicate sclerosis.

Disease Models & Mechanisms • Supplementary information

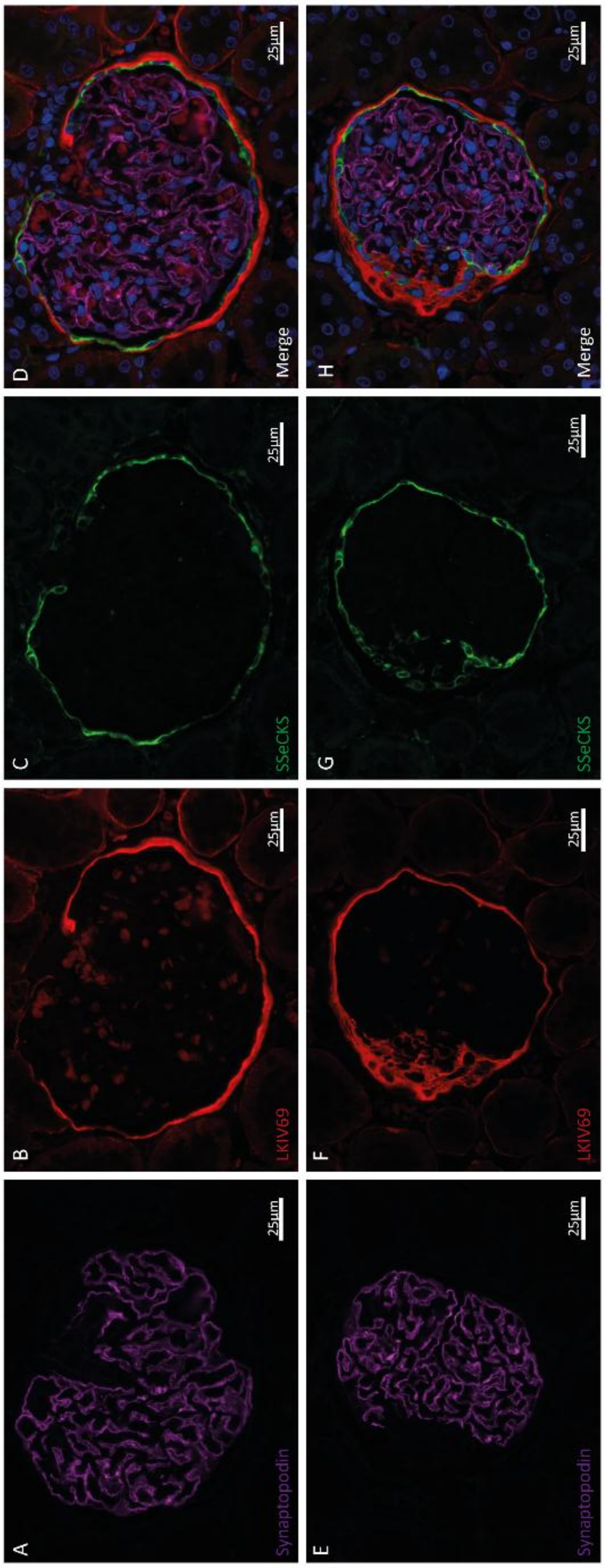

Fig. S3. LKIV69 expression represents the presence of PECs. (**A-D**) A healthy MWF rat glomerulus is shown. (**E-H**) A sclerotic MWF rat glomerulus is shown. Synaptopodin (violet), LKIV69 (red), SSeCKS (green) and DAPI (blue) signalling is depicted. The signal of SSeCKs seen in the cell body of the PECs overlap with the matrix signal LKIV69.

Disease Models & Mechanisms • Supplementary information

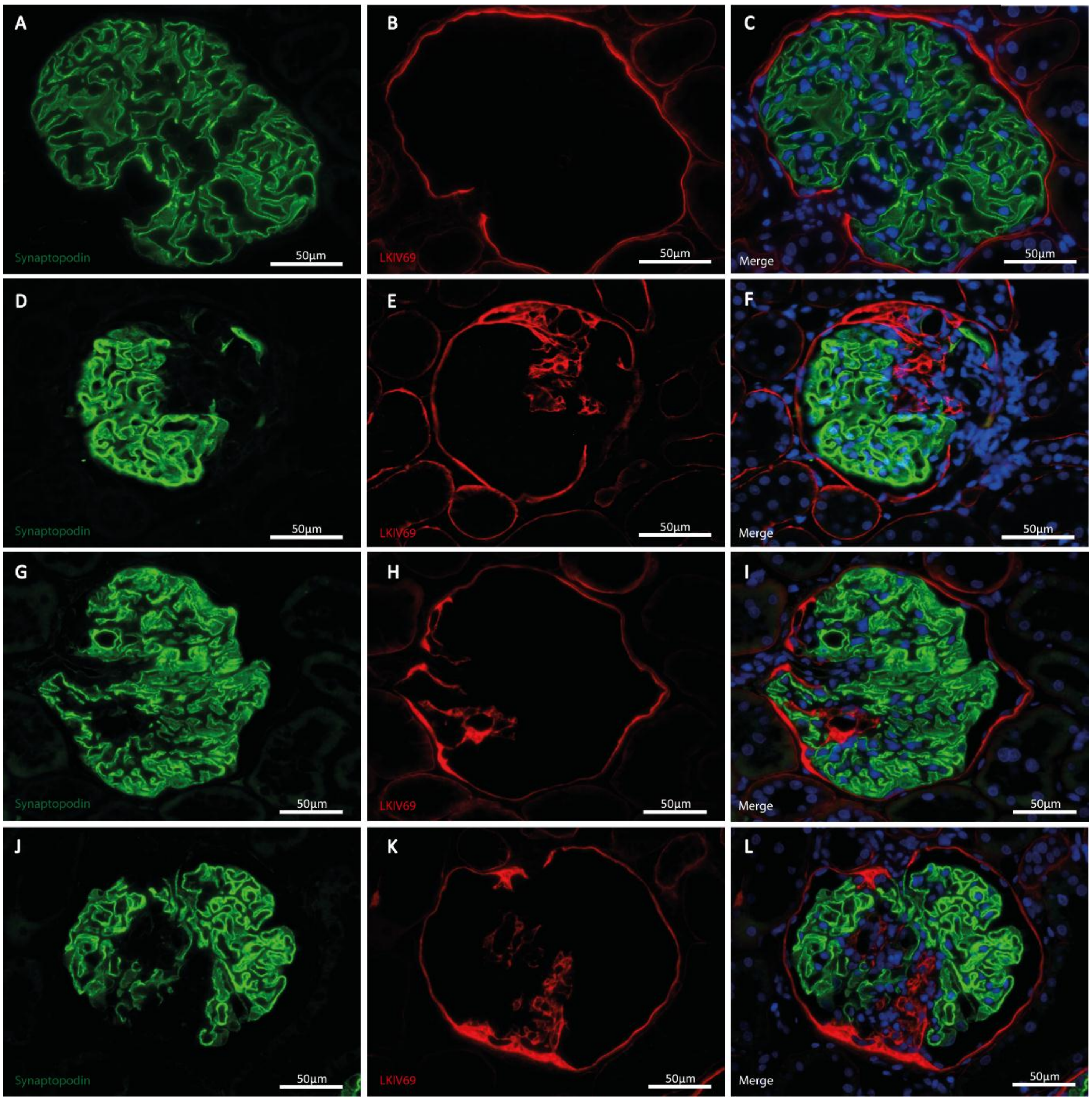

Fig. S4. Connections between PEC matrix and the glomerular tuft at sclerotic areas are formed- single channels. (**A**, **D**, **G**, **J**) The green colour reflects the synaptopodin signal seen in podocytes. (**B**, **E**, **H**, **K**) The red colour reflects the LKIV69 matrix deposited by the glomeruli. (**C**, **F**, **I**, **L**) Merge images of the LKIV69 and synaptopodin signal, including DAPI staining (blue).Fig. S3.

Disease Models & Mechanisms • Supplementary information

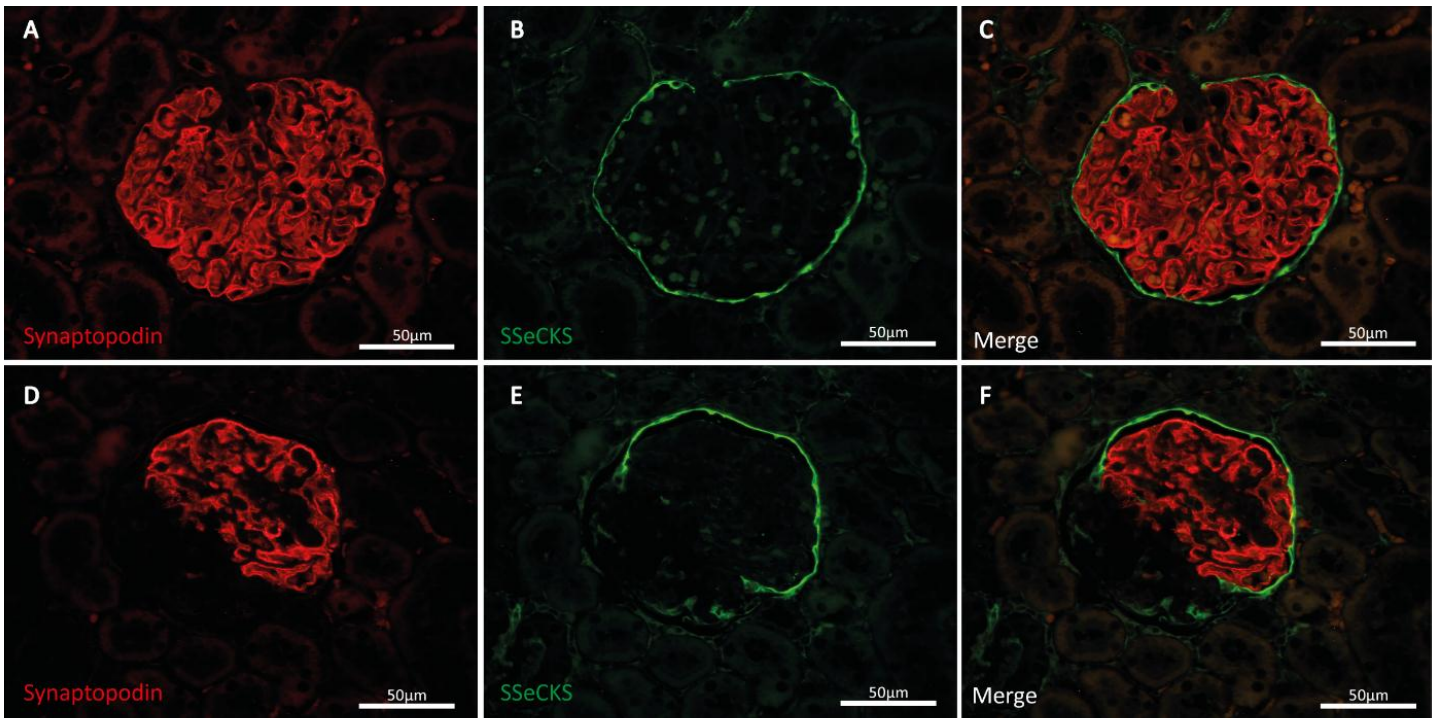

Fig. S5.Connections between PECs and the glomerular tuft at the sclerotic areas are formed, SSeCKS and synaptopodin single channels. (**A-C**) A non-affected glomerulus and (**D-F**) a sclerotic glomerulus of MWF rat tissues are depicted. Synaptopodin (red, A, D), SSeCKS (green, B, E) and merge images (C, F) are shown

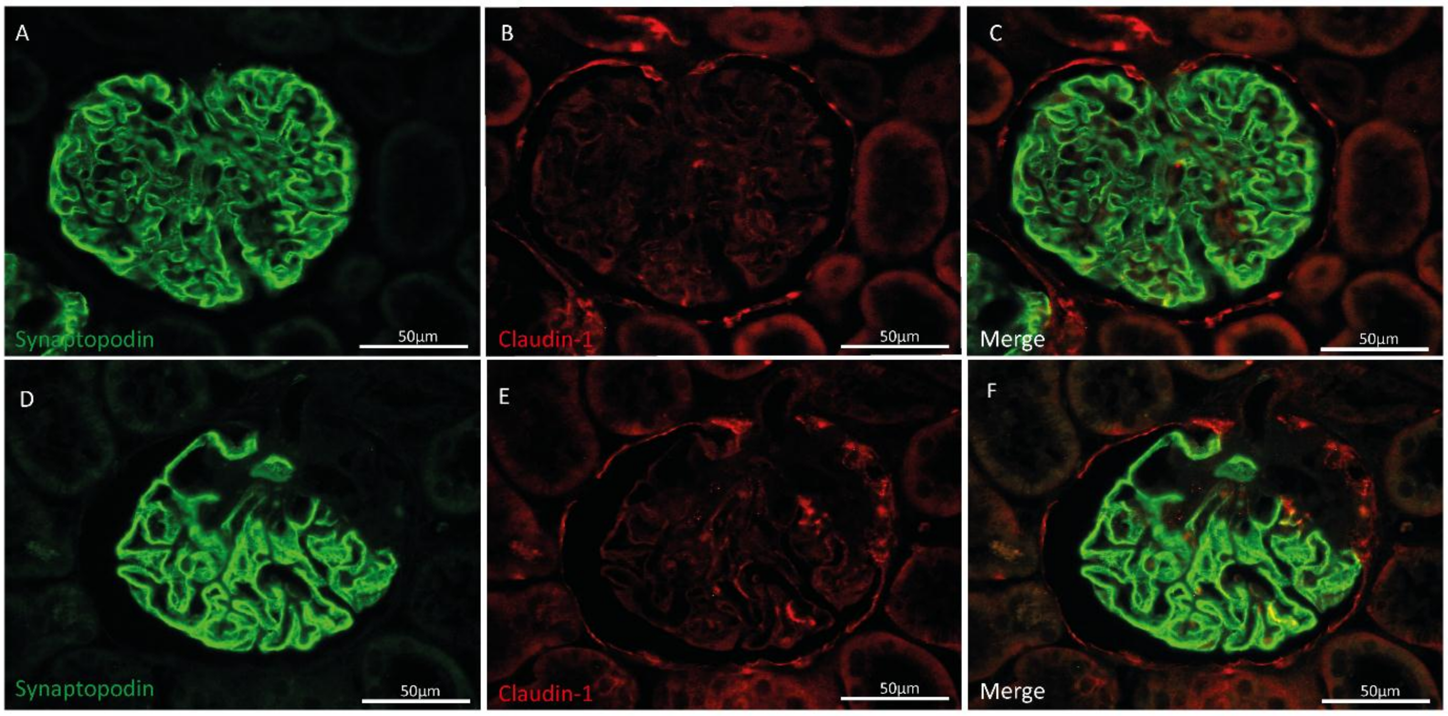

Fig. S6. Connections between PECs and the glomerular tuft at the sclerotic areas are formed, claudin-1 and synaptopodin- single channels. (**A-C**) A healthy MWF rat glomerulus is shown. (**D-F**) A sclerotic MWF rat glomerulus is shown. Synaptopodin (green, A,D) and claudin-1 (red, B, E) expression is depicted. (C,F) Merge images are shown. Contact points between claudin-1 and synaptopodin signal can be seen.

Disease Models & Mechanisms • Supplementary information

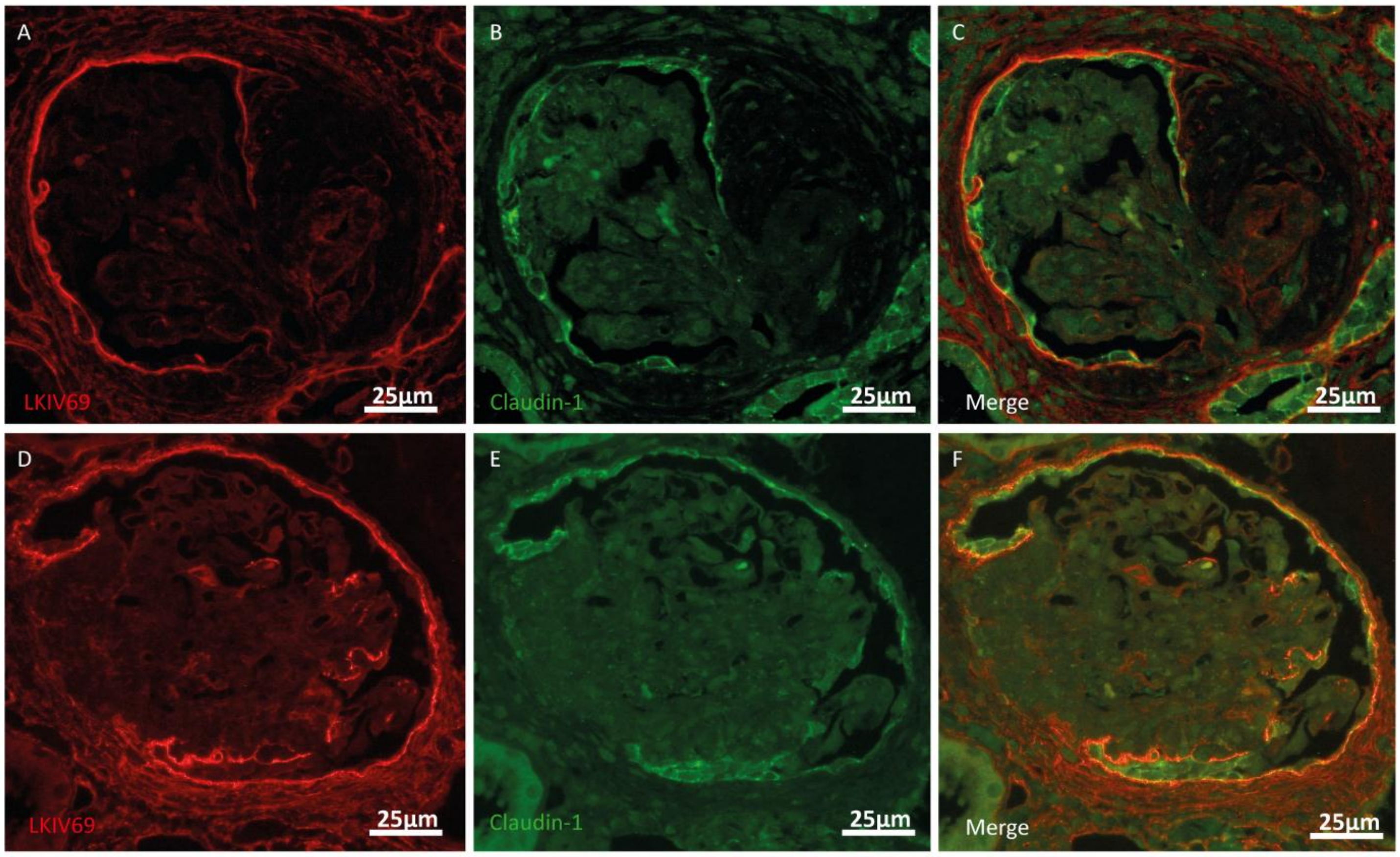

Fig. S7. In humans PECs form matrix layers between uninvolved and sclerotic glomerular segments- single channels. (**A-F**) Sclerotic human glomeruli are shown. LKIV69 (red, A, D) and claudin-1 (green, B, E) expression is depicted. (C,F) Merge images are shown.

Disease Models & Mechanisms • Supplementary information

Table S1. Antibodies used in immunostainings

Primary antibody	Dilution
SSeCKS (pAb, made in rabbit, kindly provided by Prof E. Gelman)	1:500
Synaptopodin (pAb, sc-21,537, lot L0414, Santa Cruz)	1:400
CD44 (clone 8E2, mAb, lot 2, 5640S, Cell Signaling)	1:100
Claudin-1 (pAb, ab15098, lot GR282937-7, Abcam)	1:100
ANXA3 (pAb, HPA013398, lot A101914, Atlas Antibodies)	1:200
Nephrin (pAb, AF4269-SP, lot ZMU02117071, R&D Systems)	1:300

For the following antibodies, validation was performed by the manufacturers: Synaptopodin (pAb, sc-21537, lot L0414, Santa Cruz), CD44 (clone 8E2, mAb, lot 2, 5640S, Cell Signalling), Claudin-1 (pAb, ab15098, lot GR282937-7, Abcam), Nephrin (pAb, AF4269-SP, lot ZMU02117071, R&D Systems).

Information on the anti-SSeCKS antibody can be found in the following paper:
- Burnworth B, Pippin J, Karna P, Akakura S, Krofft R, Zhang G, Hudkins K, Alpers CE, Smith K, Shankland SJ, Gelman IH, Nelson PJ. SSeCKS sequesters cyclin D1 in glomerular parietal epithelial cells and influences proliferative injury in the glomerulus. 2012. Lab Invest;92(4):499-510. doi: 10.1038/labinvest.2011.199. PMID: 22249313

Information on LKIV69 can be found in the following paper:
- Wijnhoven TJ, Lensen JF, Rops AL, van der Vlag J, Kolset SO, Bangstad HJ, Pfeffer P, van den Hoven MJ, Berden JH, van den Heuvel LP, van Kuppevelt TH. 2006. Aberrant heparan sulfate profile in the human diabetic kidney offers new clues for therapeutic glycomimetics. Am J Kidney Dis;48(2):250-61. doi: 10.1053/j.ajkd.2006.05.003. PMID: 16860191

Disease Models & Mechanisms • Supplementary information

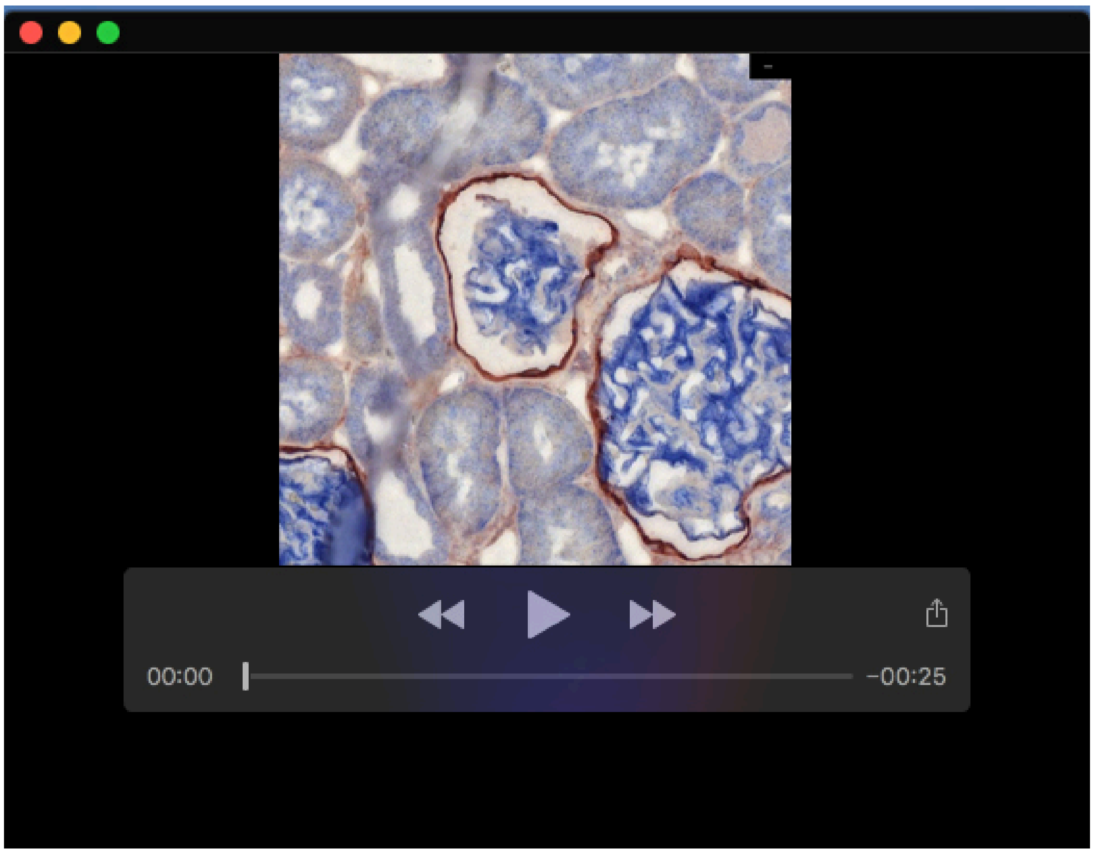

Movie 1. Stacked images of a sclerotic glomerulus. Registration of the images of the stained consecutive MWF tissue sections allowed the creation of serial images and stacking of selected glomerlui. Here, an example of a sclerotic glomerulus is given.

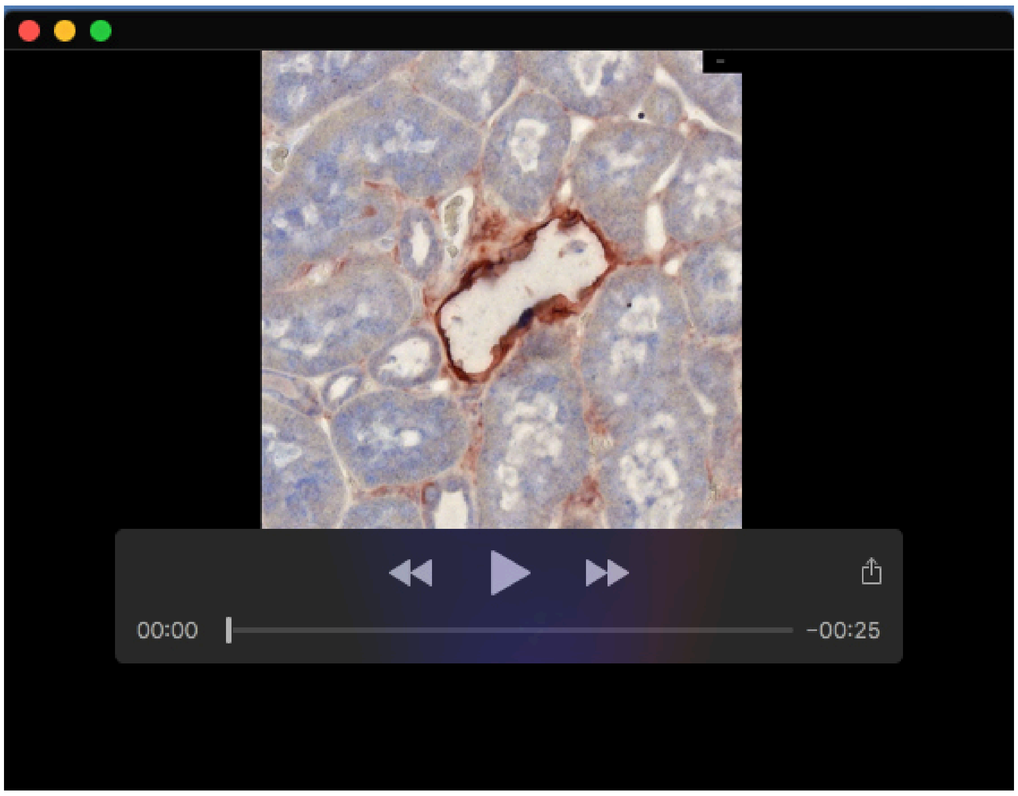

Movie 2. Stacked images of a sclerotic glomerulus. Registration of the images of the stained consecutive MWF tissue sections allowed the creation of serial images and stacking of selected glomerlui. Here, an example of a sclerotic glomerulus is given.

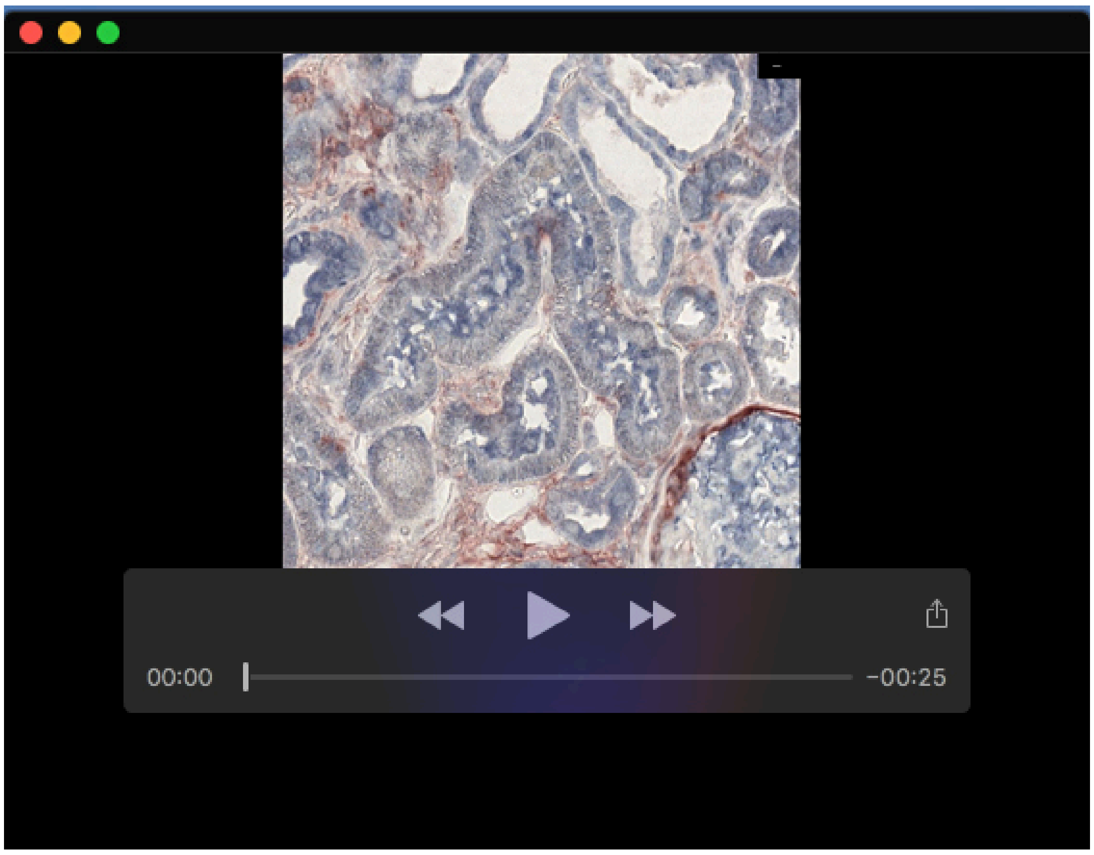

Movie 3. Stacked images of a non-sclerotic glomerulus. Registration of the images of the stained consecutive MWF tissue sections allowed the creation of serial images and stacking of selected glomerlui. Here, an example of a non-sclerotic glomerulus is given.

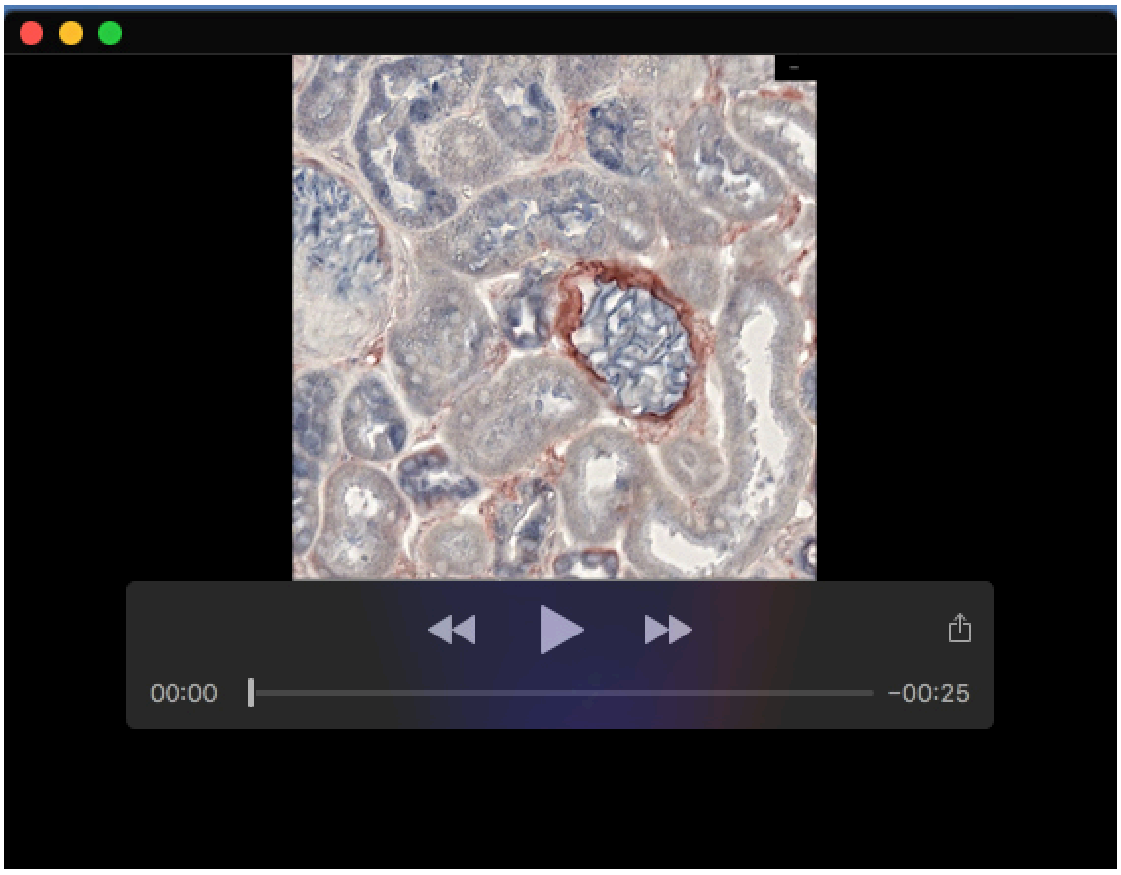

Movie 4. Stacked images of a non-sclerotic glomerulus. Registration of the images of the stained consecutive MWF tissue sections allowed the creation of serial images and stacking of selected glomerlui. Here, an example of a non-sclerotic glomerulus is given.

Disease Models & Mechanisms • Supplementary information
